# Inflammatory pain control by blocking oxidized phospholipid-mediated TRP channel activation

**DOI:** 10.1038/s41598-017-05348-3

**Published:** 2017-07-14

**Authors:** Beatrice Oehler, Katrin Kistner, Corinna Martin, Jürgen Schiller, Rafaela Mayer, Milad Mohammadi, Reine-Solange Sauer, Milos R. Filipovic, Francisco R. Nieto, Jan Kloka, Diana Pflücke, Kerstin Hill, Michael Schaefer, Marzia Malcangio, Peter W. Reeh, Alexander Brack, Robert Blum, Heike L. Rittner

**Affiliations:** 1 Department of Anesthesiology, University Hospital of Wuerzburg, Wuerzburg, Germany; 2 Institute of Clinical Neurobiology, University Hospital of Wuerzburg, Wuerzburg, Germany; 3 Institute for Physiology and Pathophysiology, Friedrich-Alexander University of Erlangen-Nuremberg, Erlangen, Germany; 4 Institute for Medical Physics and Biophysics, University of Leipzig, Leipzig, Germany; 5 Department of Chemistry and Pharmacy, Friedrich-Alexander University Erlangen-Nuremberg, Erlangen, Germany; 6 Wolfson CARD, King’s College London, Guys’ Campus, London, United Kingdom; 7 Rudolf-Boehm-Institute for Pharmacology and Toxicology, University of Leipzig, Leipzig, Germany; 8 University of Bordeaux, IBGC, UMR 5095, Bordeaux, France; 9 University of Granada, Department of Pharmacology, Granada, Spain

## Abstract

Phospholipids occurring in cell membranes and lipoproteins are converted into oxidized phospholipids (OxPL) by oxidative stress promoting atherosclerotic plaque formation. Here, OxPL were characterized as novel targets in acute and chronic inflammatory pain. Oxidized 1-palmitoyl-2-arachidonoyl-*sn*-glycero-3-phosphocholine (OxPAPC) and its derivatives were identified in inflamed tissue by mass spectrometry and binding assays. They elicited calcium influx, hyperalgesia and induced pro-nociceptive peptide release. Genetic, pharmacological and mass spectrometric evidence *in vivo* as well as *in vitro* confirmed the role of transient receptor potential channels (TRPA1 and TRPV1) as OxPAPC targets. Treatment with the monoclonal antibody E06 or with apolipoprotein A-I mimetic peptide D-4F, capturing OxPAPC in atherosclerosis, prevented inflammatory hyperalgesia, and *in vitro* TRPA1 activation. Administration of D-4F or E06 to rats profoundly ameliorated mechanical hyperalgesia and inflammation in collagen-induced arthritis. These data reveal a clinically relevant role for OxPAPC in inflammation offering therapy for acute and chronic inflammatory pain treatment by scavenging OxPAPC.

## Introduction

Hallmarks of inflammation – occurring, for example, acutely in wounds after surgery or chronically in rheumatoid arthritis - are pain and hyperalgesia. Proalgesic inflammatory mediators include prostaglandins, bradykinin, reactive oxygen species (ROS) and their downstream products^1^. Non-steroidal anti-inflammatory drugs target enzymes responsible for the formation of prostaglandins and are among the most widely used painkillers worldwide. Despite their unequivocal effectiveness serious side effects like gastric bleeding, renal failure or heart attacks restrict non-steroidal anti-inflammatory drug usage indicating the need for alternative treatment options.

In inflammation, macrophages and neutrophils are major sources of ROS^2, 3^. Oxidized phospholipids (OxPL) are generated from the plasma membrane and from circulating lipoproteins through either enzymatic or non-enzymatic mechanisms. They are highly reactive and promote pro- and anti-inflammatory pathways. In atherosclerosis OxPL support adhesion of monocytes to endothelial cells, transformation of macrophages into foam cells containing oxidized low density lipoprotein (OxLDL), plaque formation, and chemokine production^4–6^. On the other hand, OxPL block lipopolysaccharide-induced activation of toll-like receptors^7^ or simply activate toll-like receptors possibly in complexes with CD36^8^. Thus, OxPL are well known to shape the pathophysiology of atherosclerosis, but might also play a role in acute inflammatory (acute lung injury or sepsis)^9^, chronic inflammatory or neurodegenerative diseases (Alzheimer’s and Parkinson’s)^10, 11^. One of the commercially available OxPL is oxidized 1-palmitoyl-2-arachidonoyl-*sn*-glycero-3-phosphocholine (OxPAPC) which consists of a mixture of oxidized, chain-shortened phospholipids including 1-palmitoyl-2-(5-oxovaleroyl)-*sn*-glycero-3-phosphocholine (POVPC) and 1-palmitoyl-2-glutaryl-*sn*-glycero-3-phosphocholine (PGPC) as well as oxygenated phospholipids such as 1-palmitoyl-2-(5,6)-epoxyisoprostaneE_2_-*sn*-glycero-3-phosphocholine (PEIPC)^12, 13^. OxPAPC species have been previously detected in inflammation, but its role in inflammatory pain is entirely unknown^7^.

Atherosclerotic plaque formation and OxPL are reduced by apolipoprotein A-I (ApoA-I), a structural protein of the high density lipoproteins^14^. Indeed, the ApoA-I mimetic peptide D-4F reduces plaque size in early vascular lesions as well as the pro-inflammatory reactions of low density lipoproteins (LDL). Furthermore, it might improve symptoms of other autoimmune diseases such as scleroderma, collagen-induced arthritis or systemic lupus^15, 16^. A second approach to neutralize OxPL is an antibody. Using sera of ApoE-deficient mice, autoantibodies against oxidized phospholipids have been extracted of which subtype E06, also known as T15, most efficiently binds copper-oxidized LDL^17, 18^.

ROS belong to the group of pro-nociceptive mediators and are generated by resident or infiltrating leukocytes via e.g. NADPH oxidase complexes. Besides oxidation of reactive sites of proteins, particularly free sulfhydryl groups, ROS can also oxidize lipids and phospholipids in plasma membranes. ROS and their downstream products themselves are pro-nociceptive through activation of transient receptor potential vanilloid 1 (TRPV1) or ankyrin 1 (TRPA1) receptors in sensory neurons. TRPA1 and TRPV1 channels are receptors of chemical stimuli in nociceptors and act as sensors in the pain pathway^1^. TRPA1 and TRPV1 are both expressed in peptidergic nociceptors containing calcitonin gene-related peptide (CGRP) as well as in non-peptidergic isolectin 4-positive nociceptors^19, 20^. TRPA1-expressing dorsal root ganglion neurons (DRGs) overlap with TRPV1-expressing neurons, but TRPV1 is more abundantly expressed^21^. TRPV1 is activated by capsaicin, the pungent ingredient of chili peppers, and by oxidized linoleic acid metabolites like 9-hydroxy-10E- and 13-hydroxy-10E,12Z-octadecadienoic acid (9-HODE and 13-HODE) released in inflammation or after exposure to heat^22^. TRPA1 responds to endogenous and exogenous irritants like allyl isothiocyanate (AITC)^21^ and also to 4-hydroxynonenal, a downstream metabolite of OxPL found in complete Freund’s adjuvant (CFA)-induced hindpaw inflammation^3^. OxPL in turn are generated by ROS and present in inflamed tissues^9^.

Here, we report that OxPAPC is an important endogenous player in inflammatory pain. OxPAPC acts through TRPA1 and TRPV1 ion channels on nociceptors. The ApoA-I mimetic peptide D-4F or the anti-OxPL antibody E06 antagonize OxPAPC-induced hyperalgesia. Both agents constitute novel therapeutic options for the treatment of hyperalgesia in acute and chronic inflammation.

## Results

### Thermal and mechanical hyperalgesia by local OxPAPC injection in rats

**Figure 1:**
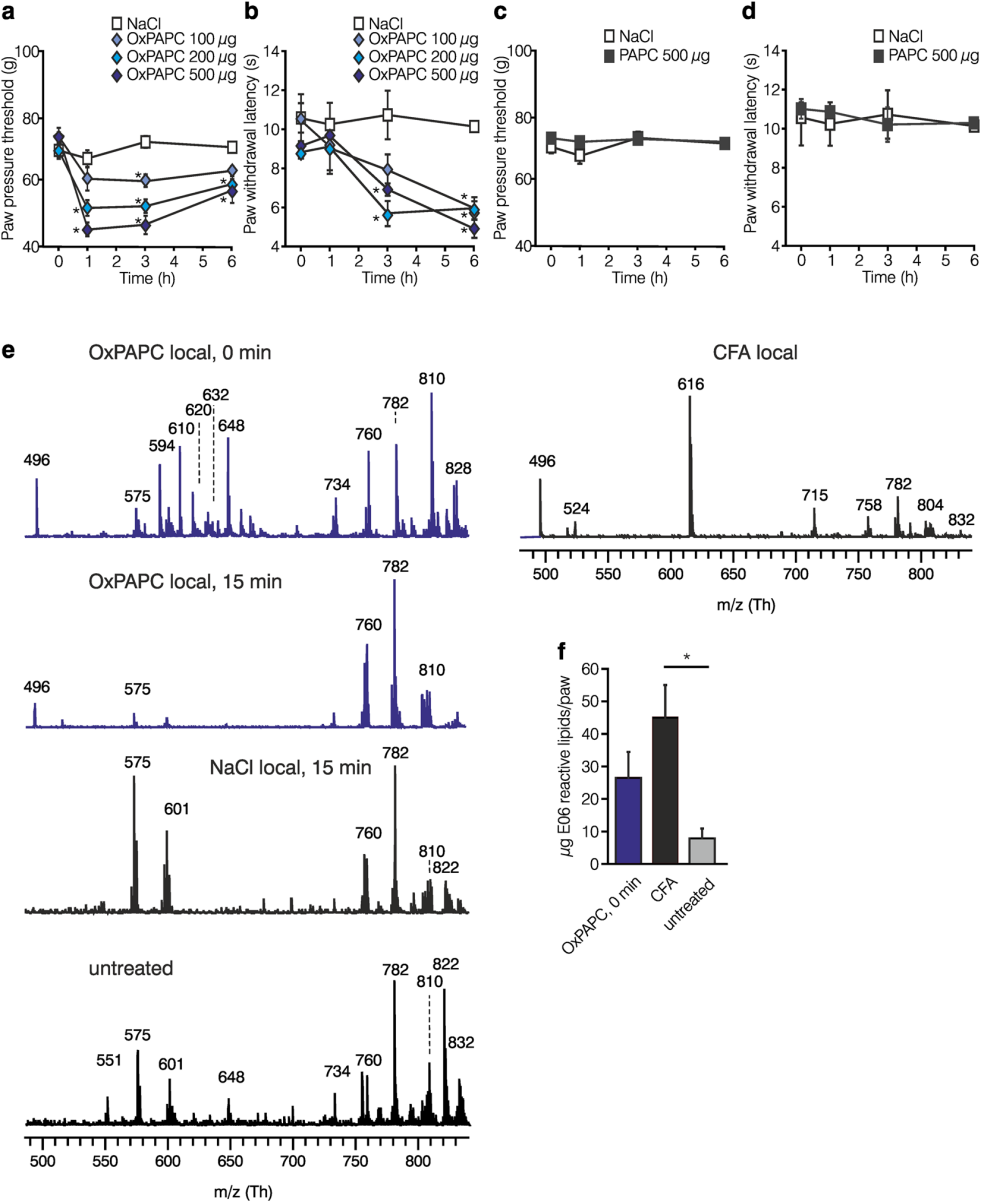
OxPAPC induces hyperalgesia in rats and is produced in inflamed tissue. (**a–d**) Pain behavior was evaluated after intraplantar injection of OxPAPC (**a**,**b**) or PAPC (**c**,**d**) by measuring mechanical (paw pressure thresholds, **a**,**c**) and thermal (paw withdrawal latencies, **b**,**d**) nociceptive thresholds before and after indicated time points (OxPAPC: 100 µg , 200 µg , and 500 µg ; PAPC: 500 µg **▪**; solvent control: 0.9% NaCl □, n = 6 Wistar rats per group, mean ± SEM, two-way RM ANOVA post hoc Holm-Sidak, *p ≤ 0.05). (**e**) Positive ion MALDI-TOF mass spectrometric analysis of chloroform tissue extracts were performed. Mass spectra are representing analyses from rat paw tissue 0 and 15 min after intraplantar injection of 500 µg OxPAPC , 3 h post intraplantar CFA injection, 15 min after intraplantar solvent, 0.9% NaCl injection, or from untreated rats (all ). Peaks are marked by *m/z* values. Scaling was set to the same intensity for the most intense peak in each measurement. Detailed assignment of the identified metabolites is given in Supplementary Table 1. Note the differences in peaks of the oxidized PAPC metabolites, lysophosphatidylcholine (LPC, *m/z* 496, 518, 524, 546), in CFA- and OxPAPC-injected compared to untreated rats. Peaks at *m/z* 575.5 and 601.5 indicate loss of one fatty acid (sodium salt) from triacylglycerols (asterisks: signals due to 2,5-dihydroxybenzoic acid matrix cluster ions, representative example, n = 3). (**f**) Quantitation of OxPAPC *in vivo* was measured by an E06-based competitive binding assay on OxPAPC-coated strips. Lipids were extracted from animals treated with OxPAPC for 0 min , CFA for 3 h , as well as untreated paws  (n = 6 per group, mean ± SEM, one-way ANOVA post hoc Holm-Sidak, *p ≤ 0.05).

As OxPAPC is generated by ROS in the inflamed tissue in other diseases we evaluated whether OxPAPC elicits hyperalgesia when injected in the rat paw. Intraplantar injection of OxPAPC dose-dependently lowered mechanical thresholds for up to 6 h with a maximal effect at 1–3 h at the dose of 500 µg (Fig. 1a). Thermal thresholds declined from 3 to 6 h after injection (Fig. 1b). Mechanical or thermal nociceptive thresholds remained unchanged in uninjected contralateral paws (Supplementary Fig. 1a,b), after local application of non-oxidized PAPC (500 µg; Fig. 1c,d, Supplementary Fig. 1c,d) or solvent control (0.9% NaCl).

### Detection of OxPAPC in CFA-induced inflammation

To evaluate a possible contribution of endogenous OxPAPC to inflammatory hyperalgesia, we examined the presence of OxPAPC in inflamed tissue compared to saline-injected or untreated controls. We used a model of localized inflammation by injection of CFA into rat’s hindpaws. Inflammation including swelling, leukocyte invasion, mechanical and thermal hyperalgesia develops within 2 h (see below;^23^).

For detection of OxPAPC species in inflamed tissue we used matrix-assisted laser desorption ionization time-of-flight mass spectrometry (MALDI-TOF MS). The commercially available OxPAPC preparation used here contained non-oxidized PAPC (*m/z* 782), 1-palmitoyl-2-(5-oxovaleroyl)-*sn*-glycero-3-phosphocholine (POVPC, *m/z* 594), 1-palmitoyl-2-glutaryl-*sn*-glycero-3-phosphocholine (PGPC, *m/z* 610) and 1-palmitoyl-2-(5,6-epoxyisoprostane E2)-*sn*-glycero-3-phosphocholine (PEIPC, *m/z* 828,), wherein the acid is about twice as abundant as confirmed by thin-layer chromatography (Supplementary Fig. 2b). *In vitro* OxPAPC itself was barely degraded while PAPC was – as expected – strongly oxidized in ambient air (Supplementary Fig. 2). In subcutaneous tissue, we detected non-oxidized PC species (*m/z* 758–832; Fig. 1e, Supplementary Table 1). Immediately after intraplantar injection of OxPAPC (0 min) several OxPAPC species including POVPC and PGPC were present, but after 15 min they disappeared and only low mass fragments of OxPAPC were detectable (e.g. *m/z* 496). After local CFA injection we also found low mass fragments of OxPAPC and, in addition, OxPAPC peaks in various intensities (e.g. *m/z* 616, POVPC).

For quantitation of OxPAPC *in vivo* we established a competitive binding assay using the E06 antibody (Fig. 1f). We measured concentrations after local treatment of CFA in rats’ hindpaws compared to rats treated with OxPAPC (500 µg/paw) as well as in untreated controls. In CFA-induced inflammation, the paw tissue contained three to five times more E06-binding OxPAPC compounds than in untreated controls (Fig. 1f).

### OxPAPC-evoked hyperalgesia and CGRP release via TRPA1 and TRPV1

**Figure 2:**
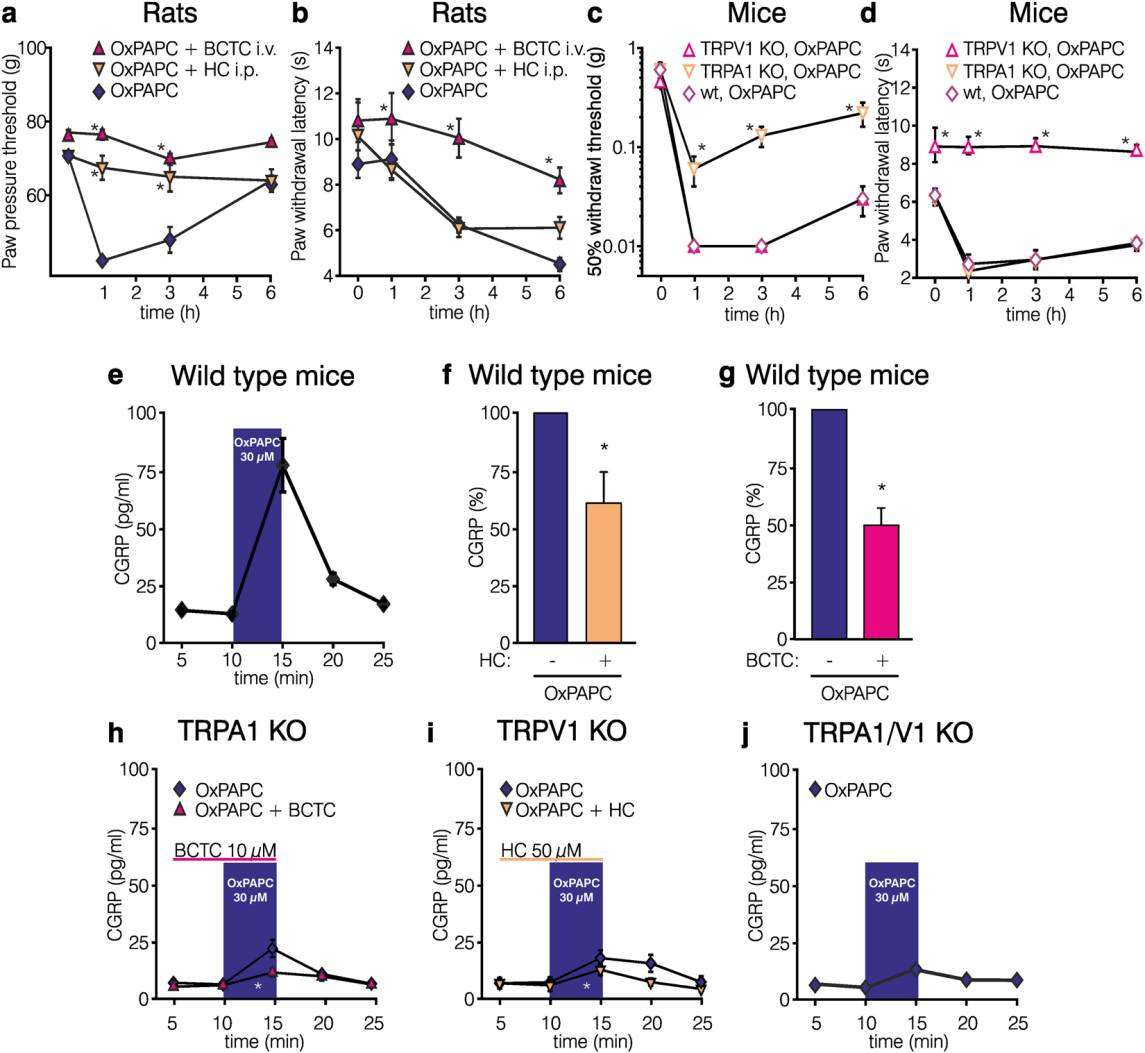
The TRP ion channels TRPA1 and TRPV1 mediate OxPAPC-induced hyperalgesia and CGRP release. Measurements of mechanical (paw pressure threshold for rats, (**a**) and the 50% threshold by von Frey test for mice, **c**) and thermal nociceptive thresholds (paw withdrawal latency, **b**,**d**) of Wistar rats (**a**,**b**) and TRPA1 or TRPV1 KO mice as well as wild type littermates (**c**,**d**) are depicted at different time points before and after intraplantar treatment with OxPAPC (500 µg in rats; ◇ 100 µg in mice). When indicated, the TRPA1 antagonist (HC-030031 , 30 mg/kg, i.p.) or the TRPV1 antagonist (BCTC , 4 mg/kg, i.v.) were co-injected with OxPAPC (all: mean ± SEM, *p ≤ 0.05; (**a**,**b**,**d**) n = 5–6, two-way ANOVA RM post hoc Holm-Sidak, (**c**) n = 6, Kruskal-Wallis ANOVA post hoc Student-Newman-Keuls). (**e–j**) OxPAPC-induced CGRP release from C57BL/6 mice hindpaw skin in response to (**e**) OxPAPC for 5 min (always 30 µM, ) and in the presence of the TRPA1 inhibitor HC-030031 (**f**, 50 µM, ) or the TRPV1 inhibitor BCTC (**g**, 10 µM, ) before OxPAPC stimulation (). (**h–j**) OxPAPC-induced CRGP release is depicted in TRPA1 KO (**h**, ), TRPV1 KO mice (**i**, ), or TRPA1/TRPV1 double KO mice (**j**, ). Residual OxPAPC-induced CGRP release in TRPA1 KO was further measured in the presence of the TRPV1 blocker BCTC (**h**, ) and in TRPV1 KO in the presence of the TRPA1 blocker HC-030031 (**i**, ). Shown are mean values ± SEM, (**e**) n = 24, (**f**–**j**) n = 8–9 mice per group, Wilcoxon matched pairs test or Mann-Whitney U-test, *p ≤ 0.05.

ROS and ROS-induced metabolites activate TRPA1 or TRPV1^21, 22^. In male Wistar rats, the TRPA1 antagonist HC-030031 completely abolished OxPAPC-induced mechanical, but not thermal hyperalgesia (Fig. 2a,b). The TRPV1 inhibitor 4-(3-chloro-2-pyridinyl)-N-[4-(1,1-dimethylethyl)phenyl]-1-piperazinecarboxamide (BCTC) reversed both, mechanical and thermal hyperalgesia (Fig. 2a,b). In TRPA1 KO mice, OxPAPC-induced mechanical hyperalgesia was significantly reduced compared to wild type littermates (Fig. 2c). Mechanical hyperalgesia was induced in both TRPV1 KO and wild type mice upon OxPAPC application. Thermal hyperalgesia was absent in TRPV1 KO (Fig. 2d), but persisted in TRPA1 KO mice and wild type littermates after intraplantar OxPAPC application. No changes were observed in contralateral paws (Supplementary Fig. 3a–f) or when antagonists were applied alone^3^.

To further analyze the functional role of TRP channels activated by OxPAPC we quantified liberation of CGRP in isolated paw skin preparations of C57BL/6 mice. OxPAPC stimulated CGRP release with a maximum effect at 30 µM (Fig. 2e; compare to Supplementary Fig. 4, 100 µM OxPAPC). CGRP liberation was inhibited by 39% with the TRPA1 antagonist HC-030031 (Fig. 2f) and by 50% with the TRPV1 blocker BCTC (Fig. 2g). When skin preparations of TRPA1 or TRPV1 KO mice were used, OxPAPC-induced CGRP release was also reduced (Fig. 2h–j, compared to Fig. 2e). In TRPA1 KO mice, a residual OxPAPC-induced CGRP release remained detectable. Antagonizing TRPV1 with BCTC could block this. Vice versa, in TRPV1 KO mice OxPAPC-induced residual CGRP liberation was blocked by the TRPA1 antagonist HC-030031 and was barely detectable in TRPA1/TRPV1 double KO mice (Fig. 2j). In summary, TRPA1 and TRPV1 are molecular mediators for OxPAPC-induced hyperalgesia and CGRP release.

### OxPAPC as an activator of TRPA1 *in vitro*

**Figure 3:**
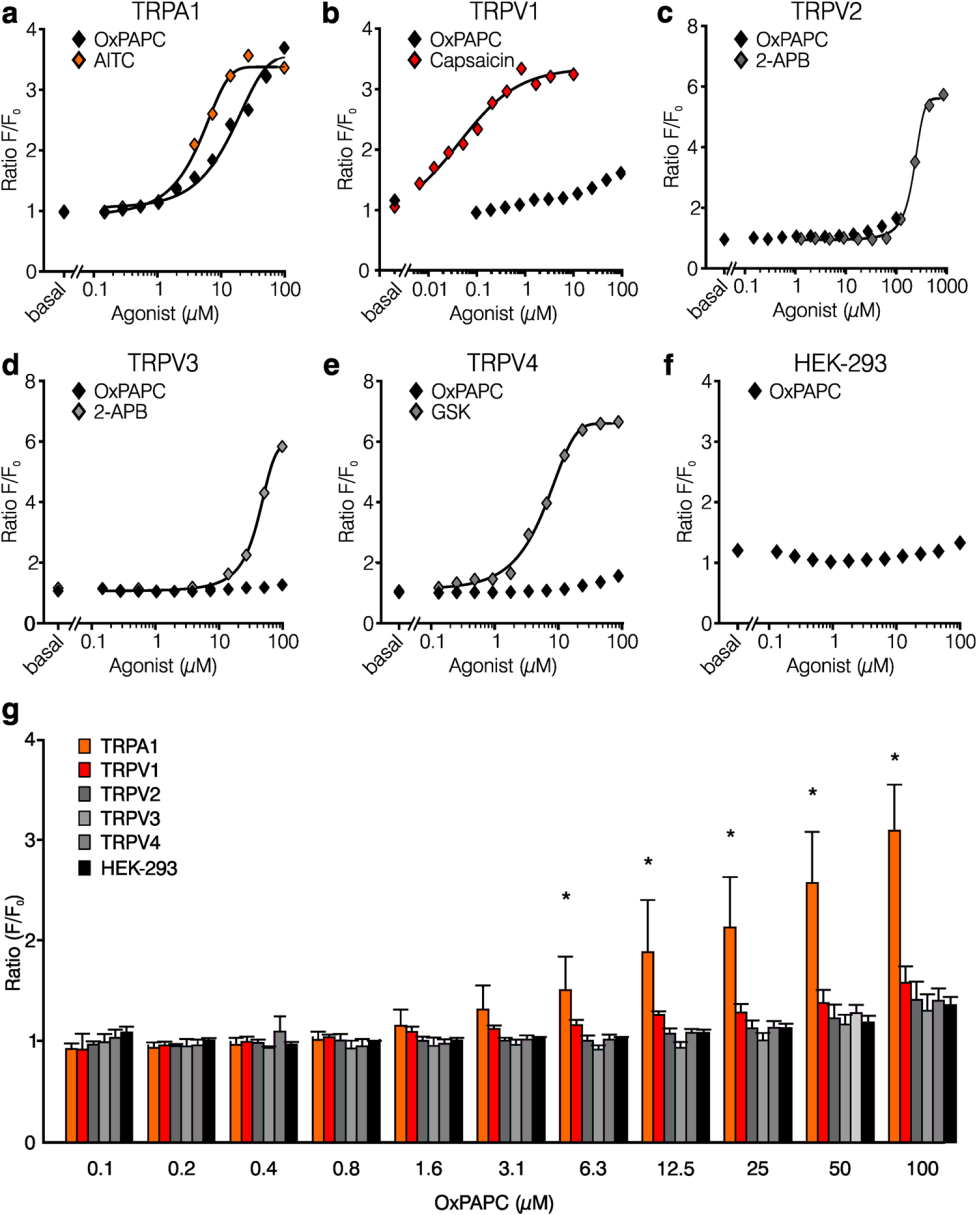
OxPAPC induces calcium transients in HEK-293 cells expressing TRPA1, but does not activate calcium influx through TRPV1-TRPV4. (**a–f**) Mean value dose-dependency curves of maximal relative changes in Fluo-4/Ca^2+^ fluorescence (ratio F/F_0_) are shown after OxPAPC stimulation of HEK-293 cells stably expressing either TRPA1 (**a**), TRPV1 (**b**), TRPV2 (**c**), TRPV3 (**d**), or TRPV4 (**e**). Untransfected HEK-293 cells served as control (**f**). OxPAPC was used at concentrations ranging from 50 nM to 100 µM (♦). For standardization, corresponding TRP agonists were used in equimolar concentrations (**a**: TRPA1: AITC ; **b**: TRPV1: capsaicin ; **c**,**d**: TRPV2, TRPV3: 2-APB (2-aminoethoxydiphenyl borate) ; **e**: TRPV4: GSK2193874 , GSK). (**g**) Summary graph of data shown in **a–f**. n = 3 of quadruple measurements, mean ± SD; two-way ANOVA RM post hoc Holm-Sidak, *p ≤ 0.05.

**Figure 4:**
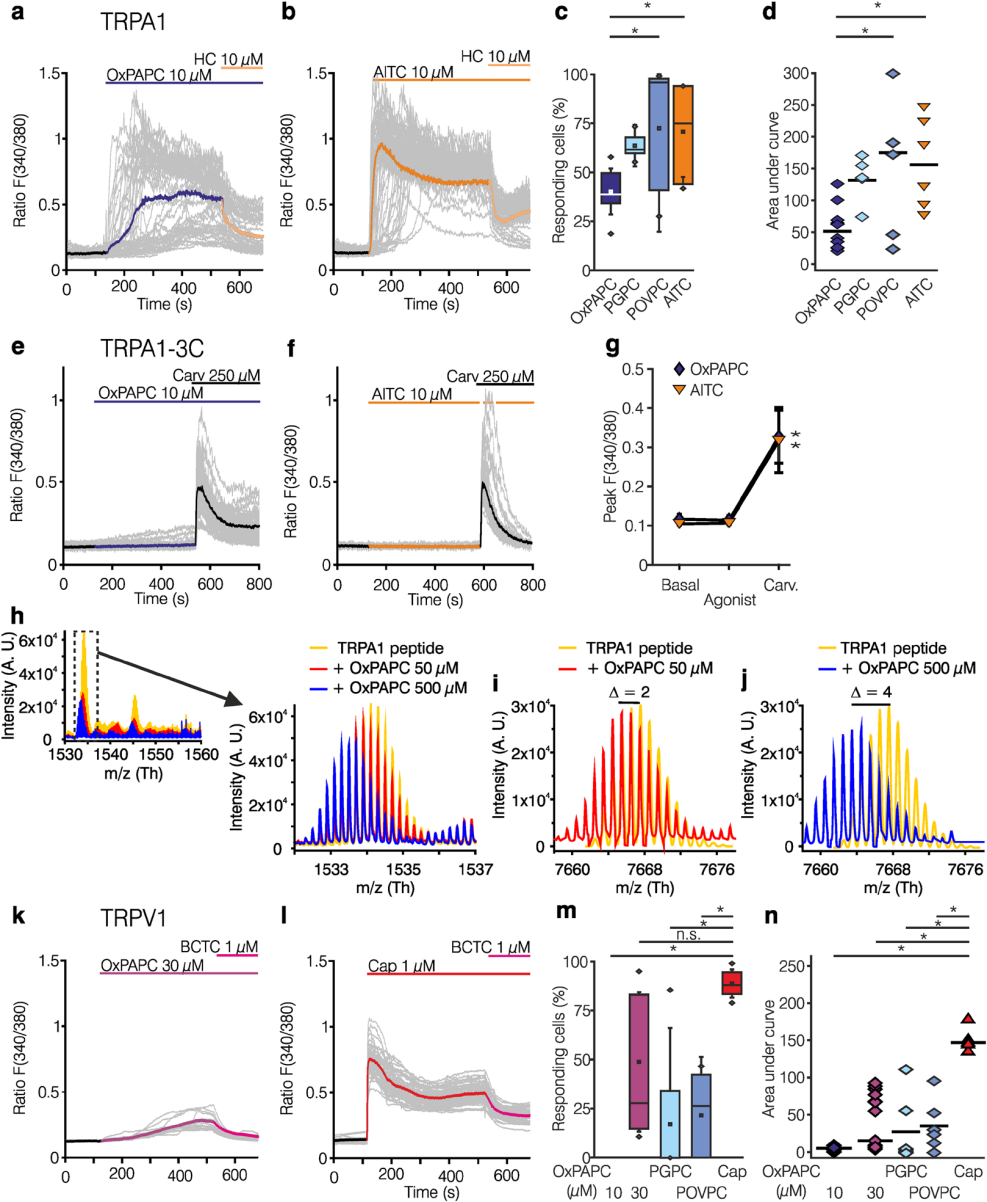
OxPAPC activates TRPA1 via cysteine residues while causing modest TRPV1 activation in transfected HEK-293. (**a–d**) Fura-2/AM-based single cell calcium imaging (ratio F(340/380)) after activation of HEK-293_TRPA1_ by OxPAPC (, 10 µM, **a**) or AITC (, 10 µM, **b**), followed by incubation with the TRPA1 antagonist HC-030031 (). Grey lines depict individual cells, the colored line an average of 100 cells. (**c**,**d**) Summary graph: The number of reacting HEK-293_TRPA1_ cells (**c**) and the AUC (**d**) compared between AITC (), OxPAPC (), PGPC () and POVPC (, all 10 µM). (**e–g**) Ca^2+^ transients were determined in HEK-293 transiently transfected with TRPA1-3C mutants lacking cysteine residues (C621S/C641S/C665S) after OxPAPC (, **e**) and AITC (, **f**) followed by the cysteine-independent activator carvacrol (, Carv). (**g**) Summary graph: Peak of the mean activation induced by OxPAPC  compared to AITC . (**h**) Mass spectrometric characterization of the reaction between TRPA1 peptide and OxPAPC: the overlaid spectra of TRPA1 (control, , and OxPAPC 50 M,  or 500 M, ) are depicted. Enlarged *m/z* region showing the ions with 5+ charge with a leftward shift in OxPAPC-treated samples (). (**i**,**j**) Overlay of deconvoluted spectra displaying the actual spectrum of the control peptide () and the peptide treated with 50 M ( **i**) or 500 M ( **j**) OxPAPC. *m/z* shift of 2 (**i**) and 4 (**j**) is suggestive of one and two disulfide bond formations, respectively. (**k–n**) Relative changes of [Ca^2+^]_i_ of HEK-293_TRPV1_ by OxPAPC (, in higher concentrations, 30 µM, **l**) or the TRPA1 agonist capsaicin (, Cap, **l**) followed by the TRPV1 antagonist BCTC (). (**m**, **n**) The summary graph of OxPAPC, PGPC, POVPC (each 10 µM), and capsaicin (1 µM) stimuli reveals two populations of HEK-293_TRPV1_ activated by the higher concentration of OxPAPC (), PGPC (), or POVPC () in contrast to a homogenous activation with capsaicin (). (**c**,**d**,**m**,**n**: n = 5–15 separate experiments, boxplot: median ± SD, outlier; scatter blot: median; t-test or one-way ANOVA post hoc Holm-Sidak *p ≤ 0.05; (**e/f**) n = 3 separate experiments, two-way ANOVA RM post hoc Holm-Sidak, *p ≤ 0.05).

To characterize the impact of OxPAPC on different TRP channel family members, we performed calcium imaging-based screening of recombinant TRP channels expressed in HEK-293 cells in a fluorescence imaging plate reader assay (Fig. 3a–f). Characteristic agonists for each TRP channel served as a reference. OxPAPC was applied at concentrations ranging from 0.05–100 µM. OxPAPC-induced calcium influx was prominent in hTRPA1-transfected HEK-293 cells already at concentrations of ≥6 µM (Fig. 3a,g). In this assay, OxPAPC neither activated recombinant TRPV2, TRPV3, or TRPV4 nor untransfected HEK-293 cells, but weakly stimulated recombinantly expressed TRPV1 at higher OxPAPC concentrations (Fig. 3b,g). Next, we created a HEK-293 cell line stably expressing hTRPA1 (HEK-293_TRPA1_) and performed single cell ratiometric calcium measurements to investigate the characteristics of TRPA1 activation by OxPAPC (Fig. 4a–g). In HEK-293_TRPA1_ OxPAPC evoked a robust and sustained increase in intracellular calcium. However, in comparison to TRPA1-mediated calcium influxes evoked by the TRPA1 agonist AITC, OxPAPC-induced calcium influx was temporally delayed at equimolar concentrations, in particular in a subpopulation of cells (Fig. 4a,b). Acute and delayed activation kinetics could best be distinguished in single traces of OxPAPC responses. In addition, the number of cells responding to an OxPAPC stimulus was lower in comparison to AITC stimulation (Fig. 4c). Calcium amplitudes (ratio F(340/380)) of cells with a pronounced OxPAPC-induced response were comparable with AITC-induced responses, however, as less cells were reactive in case of OxPAPC stimulation, the overall potency of OxPAPC was lower (Fig. 4a–d). Specific components of OxPAPC, such as PGPC and POVPC, also activated TRPA1-induced calcium influx. In comparison to OxPAPC, the number of responding cells and the area under curve were slightly increased when all reagents were compared at equimolar concentrations (Fig. 4c,d). The TRPA1 inhibitor HC-030031 (Fig. 4a,b) immediately blocked both, OxPAPC- and AITC-induced activation of TRPA1. Neither OxPAPC nor AITC stimulated control HEK-293 cells (Supplementary Fig. 5a).

The effect induced by OxPAPC suggests the potential involvement of ROS generation. Ascorbic acid (vitamin C), an antioxidant, only modestly but not significantly reduced the OxPAPC-induced activation of HEK-293_TRPA1_ (Supplementary Fig. 6a,b). In HEK-293_TRPA1_ cells loaded with dihydroethidium^24^, an oxidation-sensitive dye, the integrated fluorescence intensity rose significantly after treatment with carbonyl cyanide-4-(trifluoromethoxy)phenylhydrazone (FCCP, positive control)^25^. FCCP elicited intracellular ROS production by cellular stress due to uncoupling the ATP synthesis. After treating the cells with OxPAPC no changes were detectable during 30 min incubation time (Supplementary Fig. 6c).

TRPA1 is a polychemoreceptor^21^ and carries three N-terminal intracellular cysteines responsible for electrophilic binding of some TRPA1 agonists such as AITC^26^. To prove that these intracellular residues are involved in OxPAPC-induced effects via TRPA1, a TRPA1 mutant was investigated. In this TRPA1 mutant (hTRPA1-3C) three critical cysteines were replaced by serine (C621S/C641S/C665S)^27^. Indeed, OxPAPC and AITC failed to activate hTRPA1-3C. In contrast, carvacrol, a TRPA1 agonist, which acts through a non-electrophilic binding site in the TRPA1 receptor, still caused calcium influx, thus verifying that the TRPA1 ion channel function was intact (Fig. 4e–g). However, as the OxPAPC compounds PGPC and POVPC are not electrophilic^4^, this experiment also suggests that other mechanisms than electrophilic interaction with these critical cysteine residues contributes to TRPA1 activation by OxPL^26, 28, 29^.

To further assess the mechanism by which OxPAPC activates TRPA1 we used a model peptide of the intracellular N-terminal part of hTRPA1 (amino acids 607–670)^30, 31^, which includes three cysteine residues responsible for hTRPA1 activation by noxious stimuli^32^. No direct reaction products between OxPAPC and TRPA1 peptide were detected. Instead a dose-dependent oxidation was observed (Fig. 4h,i), with lower dose leading to formation of one disulfide bond (noticeable by leftward shift of deconvoluted mass spectrum by *m/z* = 2; Fig. 4i). Higher doses led to a more pronounced oxidation, with formation of two disulfide bonds (leftward shift of deconvoluted mass spectrum by *m/z* = 4; Fig. 4j). This indicates that oxidation of individual OxPAPC compounds with the intracellular N-terminal domain of TRPA1 might contribute to TRPA1 activation. However, for instance PGPC is an end-stage oxidation product with TRPA1 activation activity, indicating that more mechanisms than electrophilic interaction or TRPA1 oxidation contribute to OxPAPC function. OxPAPC elicited a modest and delayed activation of recombinant TRPV1. In order to attain this objective, higher concentrations of OxPAPC (30 µM instead of 10 µM) were needed. The calcium-influx through TRPV1 was blocked by BCTC (Fig. 4k–n). The number of activated HEK-293_TRPV1_ cells and the AUC of OxPAPC (10 µM) compared to capsaicin-activated HEK-293_TRPV1_ was significantly lower (Fig. 4m,n). A substantial proportion of HEK-293_TRPV1_ cells could not be activated even with a higher concentration of OxPAPC, as well as PGPC and POVPC (Fig. 4m,n). In summary, *in vitro* reconstitution showed that OxPAPC activates TRPA1 (see also^33^) and to a lesser extend TRPV1.

### OxPAPC evokes currents in HEK-293_TRPA1_ and HEK-293_TRPV1_

**Figure 5:**
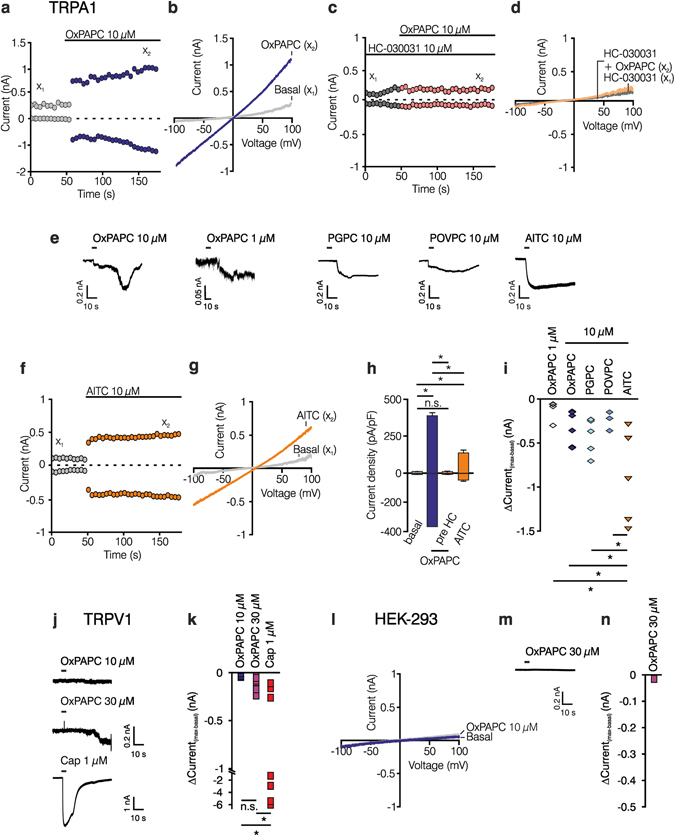
OxPAPC-evoked non-selective currents in HEK-293_TRPA1_ and HEK-293_TRPV1_ cells. (**a–e**) Representative whole cell patch clamp recording of a HEK-293_TRPA1_ after application of 10 µM OxPAPC (**a**,**b** ) or after application of 10 µM OxPAPC to cells pre-incubated in 10 µM HC-030031 (**c**,**d** ). Data in (**a**/**c**) were extracted from voltage ramps such as in (**b**/**d**) and depict currents at ± 80 mV. (**b**,**d**) Current/voltage ramps for basal (x_1_) and OxPAPC-induced (, x_2_) currents at the time points indicated in (**a**/**c**). (**e**) Recordings of TRPA1 currents upon stimulation with OxPAPC (10 µM, 1 µM), PGPC (10 µM), and POVPC (10 µM) measured at −60 mV voltage clamp compared to AITC (10 µM). (**f**,**g**) Representative whole cell patch clamp recording of HEK-293_TRPA1_ after application of 10 µM AITC (**f**, ) and current/voltage ramps (**g**) taken from indicated time point. (**h**) Statistical analysis of current densities at ± 80 mV before and up to 10 min after stimulation with OxPAPC (), as well as after blockade of OxPAPC-induced currents with HC-030031 (). For comparison, current density after activation with AITC () is displayed (n = 3 separate experiments per group, mean ± SD, one-way ANOVA post hoc Holm-Sidak *p ≤ 0.05). (**i**) Statistical analysis of ΔCurrent(_max-basal_) within 2 min upon stimulation with OxPAPC ◇**/** , PGPC , POVPC  or AITC . (**j**,**k**) Current traces and statistical analysis of HEK-293_TRPV1_ upon stimulation with 10 µM or 30 µM OxPAPC or capsaicin (1 µM). (**l**–**n**) Current-voltage ramp, current traces and ΔCurrent(_max-basal_) evoked by 30 µM OxPAPC () in parental HEK-293 cells are displayed. Voltage was clamped at −60 mV in current trace analysis and maximum was taken within 2 min after OxPAPC application. (**h**,**i**,**k**,**n**) n = 5–8, mean ± SEM, one-way ANOVA post hoc Holm-Sidak *p ≤ 0.05).

In whole cell patch clamp experiments (Fig. 5) HEK-293_TRPA1_ and HEK-293_TRPV1_ cells as well as the parental control cell line were stimulated with OxPAPC (1, 10 and/or 30 µM). Measurements on HEK-293_TRPA1_ were performed in calcium-free extracellular bath solution to prevent calcium-induced desensitization of TRPA1^34^. In addition, 50% of sodium ions were replaced by N-methyl-D-glucamine to limit OxPAPC-evoked inward currents of TRPA1^35^. OxPAPC evoked an activation of large TRPA1 currents in HEK-293_TRPA1_ cells (Fig. 5a,b,e), which were absent in the presence of HC-030031 (10 µM; Fig. 5c,d) and in the parental control cell line (Fig. 5l–n). Application of voltage ramps after addition of OxPAPC revealed a linear current voltage relationship (Fig. 5b). Furthermore, TRPA1-related currents were recorded after stimulation with 1 µM and 10 µM OxPAPC as well as PGPC, POVPC and AITC (10 µM each; Fig. 5e). Here, agonists were applied to the cell by a stimulation pipette. For comparison, current/time courses and current/voltage ramps were measured in HEK-293_TRPA1_ cells stimulated with AITC (10 µM; Fig. 5f,g). Current densities were calculated by taking the peak currents 2–10 min upon stimulation with OxPAPC, OxPAPC in the presence of HC-030031 or AITC (Fig. 5h). Analysis of the onset of TRPA1-mediated currents evoked by OxPAPC revealed a smaller current within the first 2 min compared to AITC (Fig. 5i) confirming a delayed activation kinetic of TRPA1 by OxPAPC (Fig. 5e). In HEK-293_TRPV1_ only a non-significant, delayed shift of the current could be induced by 3-fold higher concentrations of OxPAPC compared to capsaicin (Fig. 5j,k). In parental control cells, no current was observed after stimulation with either 10 µM (Fig. 5l) or 30 µM OxPAPC (Fig. 5m,n). Experiments on HEK-293_TRPV1_ and parental cells were performed in buffer containing 100% sodium chloride and calcium chloride. It has been shown that cytosolic inorganic polyphosphates support the activation of TRPA1 by pungent chemicals such as AITC. For this reason, we added polytriphosphate (PPP_i_) to the intracellular solution and performed whole-cell patch clamp recording of HEK-293_TRPA1_ cells. Local application of AITC, OxPAPC, or PGPC induced long-lasting and stable inward currents for up to 3 min (Supplementary Fig. 5b).

### Intracellular calcium increase in native DRG neurons by OxPAPC

**Figure 6:**
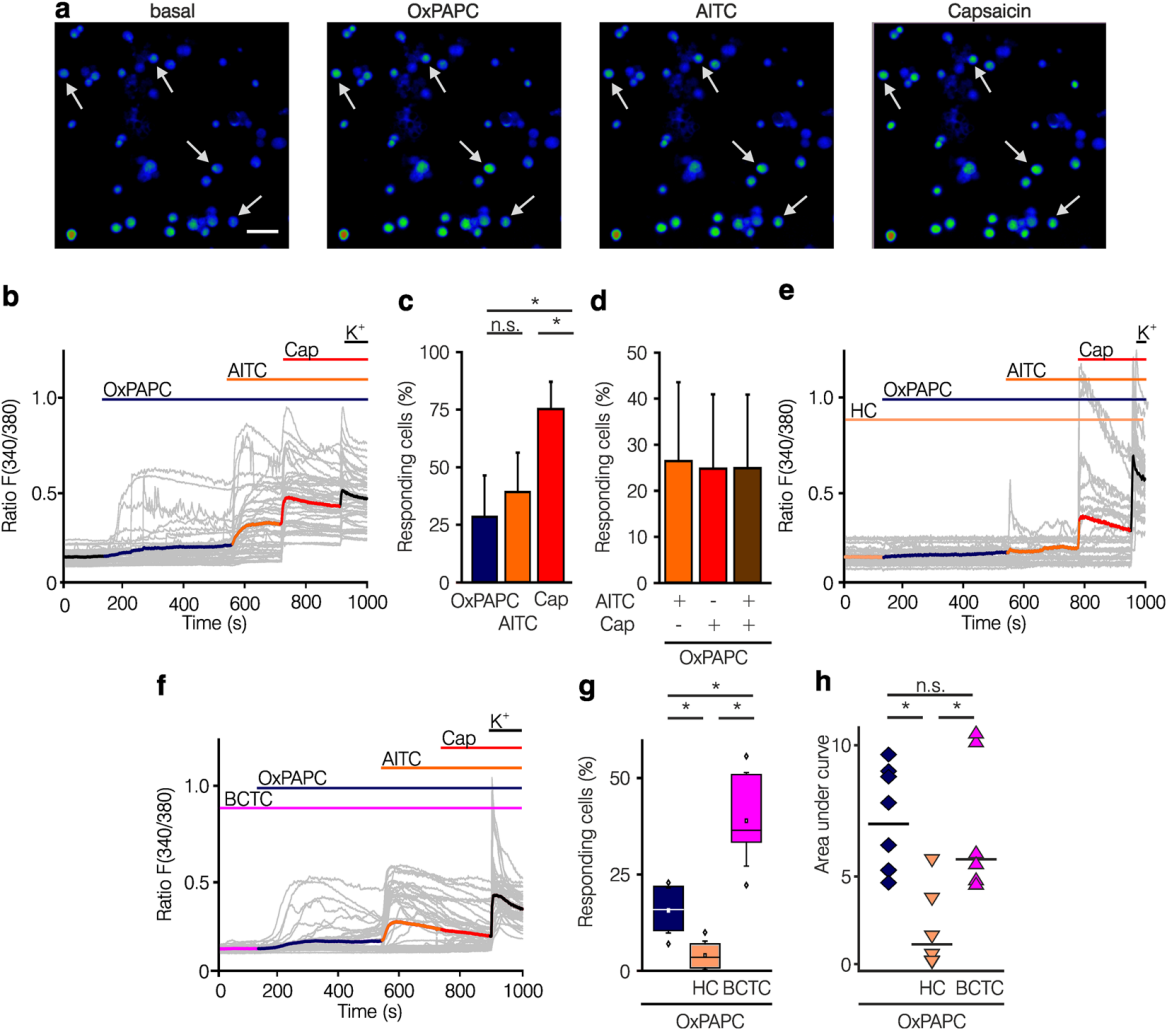
OxPAPC elicits TRPA1-dependent increase in (Ca^2+^)_i_ in cultured sensory DRG neurons of mice. (**a**) Pseudocolored images illustrating a series of (Ca^2+^)_i_ responses evoked by consecutive application of OxPAPC (, 10 µM), AITC (, 10 µM), and capsaicin (, 1 µM) measured with Fura-2 in mouse DRG neurons, in comparison to the start situation (basal). Arrows point to neurons responding to OxPAPC, the TRPA1 agonist AITC and the TRPV1 agonist capsaicin (scale: 100 µm) followed by 90 mM potassium chloride (). (**b**) Shown are curves of relative changes in (Ca^2+^)_i_, as described above in a. Grey traces indicate single cell calcium responses, ratio F(340/380). Colored line: average of 50 cells. (**c**) Comparison of (Ca^2+^)_i_ responses in DRG neurons activated by OxPAPC , AITC , and capsaicin  stimulation. (**d**) Displayed is the quantification of OxPAPC-activated DRGs after further with AITC , capsaicin , or AITC plus capsaicin  calculated by the number of responding cells. Pre-incubation of DRG neurons with the TRPA1 antagonist HC-030031 (, **e**) or the TRPV1 antagonist BCTC (, **f**) for 5 min before application of OxPAPC () is depicted. (**g**,**h**) Summary results: Inhibition of the TRPA1 antagonist HC-030031 () is compared to BCTC () analyzing the number of OxPAPC () reacting DRG neurons (**g**) and the AUC (**h**) of OxPAPC-induced calcium responses. n = 6–8 separate experiments, mean ± SD, one-way ANOVA post hoc Holm-Sidak, *p ≤ 0.05.

Next, we wanted to determine whether OxPAPC was also able to stimulate native TRPA1 in cultured sensory neurons. For this, dissociated DRG neurons of adult C57BL/6 mice and Wistar rats were used for calcium imaging experiments. Stimulation of DRG neurons with OxPAPC, AITC or capsaicin increased intracellular calcium levels (Fig. 6, Supplementary Fig. 7b). Mouse and rat neurons activated by OxPAPC also responded to AITC and capsaicin, while only few neurons were solely responsive to AITC or capsaicin (Fig. 6a–d, Supplementary Fig. 7a,b). When DRG neurons were first stimulated with AITC, OxPAPC failed to boost an increase in cytosolic calcium ion concentrations, suggesting desensitization of TRPA1 after AITC stimulation (Supplementary Fig. 7a). Pre-incubation of DRG neurons with the TRPA1 antagonist HC-030031 completely blocked the OxPAPC-induced calcium influx (Fig. 6e) while pre-incubation with the TRPV1 antagonist BCTC only diminished OxPAPC-induced activation (Fig. 6f). The number of responding cells and the AUC were significantly reduced by HC-030031, both by 25%. In contrast, BCTC rather increased the number of reacting cells and did not reduce the AUC (Fig. 6g,h). These data indicate that TRPA1 is the preferential direct target of OxPAPC in cultured adult DRG neurons of mice and rats.

### E06, a monoclonal antibody, interferes with OxPAPC function *in vitro* and *in vivo*

**Figure 7:**
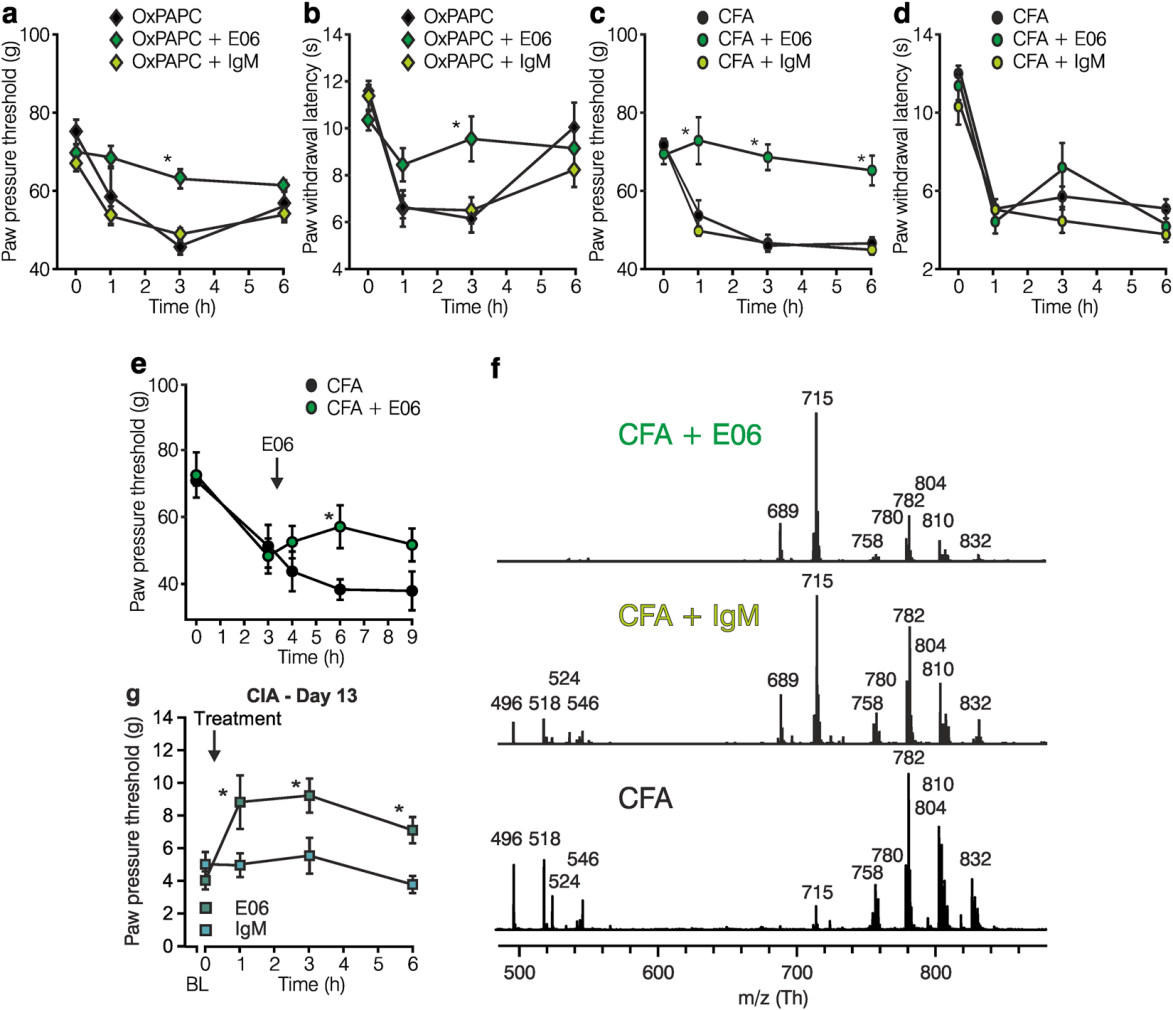
E06 mAb, an antibody against oxidized phospholipids, blocks OxPAPC- as well as CFA-induced hyperalgesia and reduces OxPL fragments in inflamed paw tissue. Mechanical (**a**,**c**) and thermal (**b**,**d**) nociceptive thresholds were determined before (0 h) and after intraplantar injection of OxPAPC (**a**,**b**;  500 µg) or CFA (**c**,**d**; ⚫ 150 µl) in Wistar rats. To counteract OxPAPC function, E06 mAb (, 1/100) or isotype control IgM Ab (, 1/100) were co-injected with OxPAPC or CFA (n = 6/group, mean ± SEM, two-way RM ANOVA post hoc Holm-Sidak, *p ≤ 0.05). (**e**) E06 mAb ( 1/100) was injected 3 h after CFA application. (**f**) Positive ion MALDI-TOF MS analysis of chloroform rat paw tissue extracts was performed in the presence of a 2,5-dihydroxybenzoic acid as matrix. Shown are mass spectra 3 h after injection of CFA together with E06 mAb (, 1/100, upper trace), CFA with IgM mAb control (, 1/100, middle trace) or CFA alone (, lower trace). Oxidized PAPC fragments are depicted (LPCs, m/z 496.3, 518.3, 524.3, 546.3) (representative example, n = 3). (**g**) In CIA, mechanical nociceptive thresholds were measured before (baseline (BL), 0 h) and after indicated time points post treatment with E06 mAb (, 1/100) or IgM isotype control (, 1/100) on day 13 (n = 8/group, mean ± SEM, two-way RM ANOVA post hoc Holm-Sidak, *p ≤ 0.05).

E06 mAb, a monoclonal IgM antibody, originally isolated from apolipoprotein-deficient mice^18^, is known to bind and neutralize OxPAPC^36^. Therefore, we asked whether E06 mAb functions as an inhibitor of OxPAPC-induced hyperalgesia *in vivo* and whether the antibody also interferes with OxPAPC-induced activation of TRPA1 *in vitro*. Intraplantar injection of OxPAPC evoked mechanical and thermal hyperalgesia (Fig. 7a,b). Hyperalgesia could be reduced by preincubation of OxPAPC with E06 mAb for 30 min before injection of the mixture into rats’ hind paws. For control, IgM isotype antibodies did not alter nociceptive thresholds. Mechanical hyperalgesia induced by injection of CFA was abrogated by co-injection of CFA with E06 mAb but not with the IgM isotype control antibody (Fig. 7c). In addition, E06 mAb reversed mechanical hyperalgesia, when injected 3 h after induction of CFA-evoked inflammation (Fig. 7e). However, E06 mAb treatment did not alter thermal CFA-induced hyperalgesia (Fig. 7d). No change in mechanical or thermal nociceptive thresholds was observed after treatment with E06 mAb *per se* without CFA or OxPAPC (Supplementary Fig. 8). Extracted lipids from the rats’ paw tissues were analyzed by MALDI-TOF MS, 3 h after CFA treatment, in presence or absence of either E06 mAb or control IgM antibodies (Fig. 7f). All samples contained non-oxidized phosphatidylcholines including PAPC metabolites. Characteristic oxidation products of PAPC (in particular *m/z* 496.3, the H^+^ adduct of lysophosphatidylcholine (LPC) 16:0) were not detectable in E06-treated animals (Fig. 7f), but remained after treatment with control IgM or in inflamed paws without treatment. In collagen-induced arthritis, a single intraplantar injection of E06 mAb on day 13 reversed mechanical hyperalgesia in contrast to IgM isotype treatment (Fig. 7g) with no change in paw edema (Supplementary Fig. 8c).

**Figure 8:**
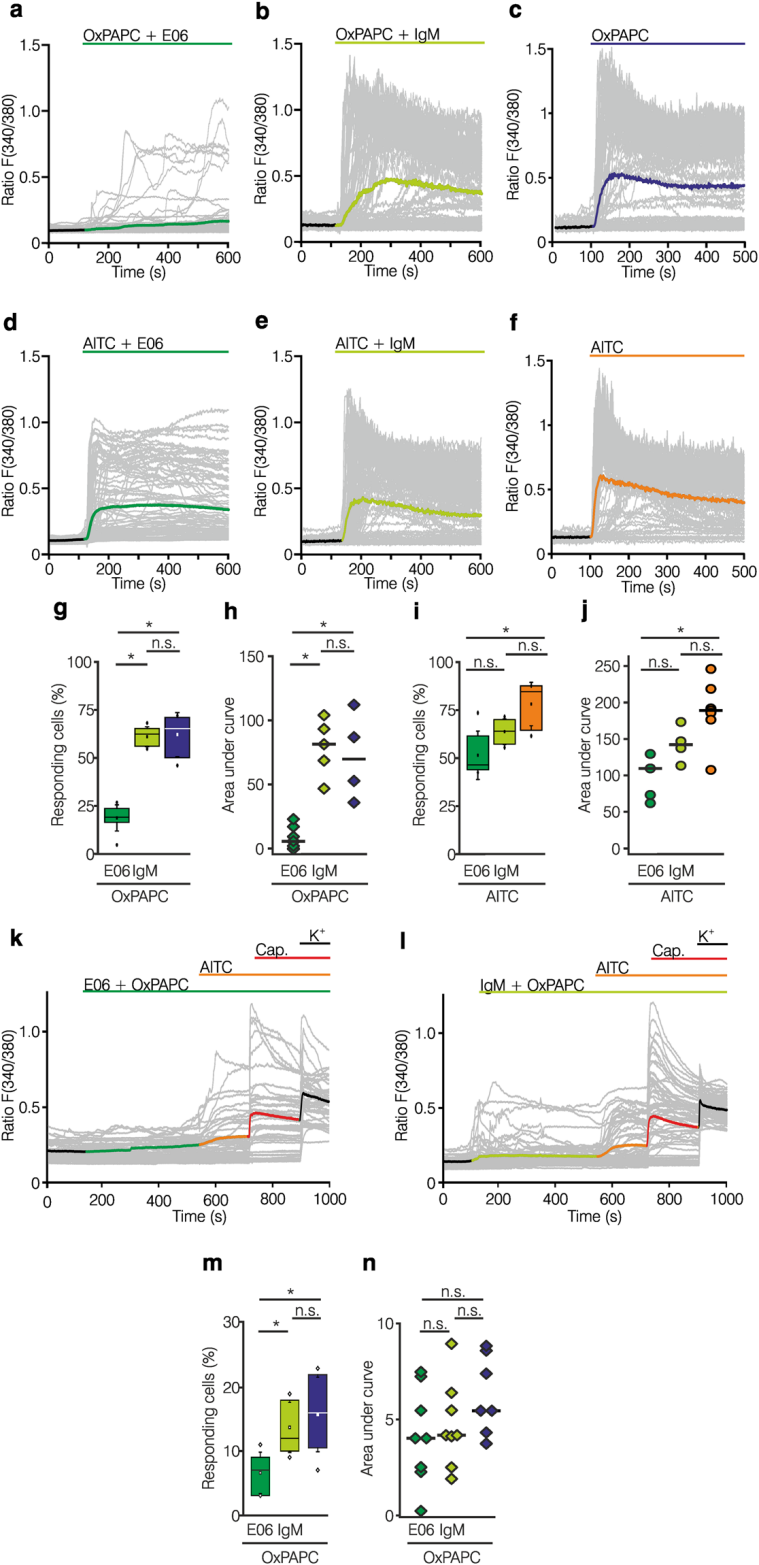
E06 mAb against OxPL inhibits OxPAPC-induced TRPA1 activation. (**a–j**) Calcium imaging experiments of HEK-293 cells expressing TRPA1 are shown. Grey traces indicate single cell calcium responses, ratio F(380/F340). Colored line: average of 100 cells. (**a**–**f**) TRPA1-mediated calcium responses were evoked by 10 µM OxPAPC ( **a–c**,**g**,**h**) or 10 µM AITC ( **d–f**,**I**,**j**) in the presence of E06 mAb ( 1/300, **a**,**d**,**g–j**) or IgM isotype control Ab ( 1/300, **b**,**e**,**g–j**). (**g–j**) OxPAPC- or AITC-mediated calcium responses and blockade by E06 mAb are summarized. Shown are percentage of reacting cells and area under curve (AUC) analysis of calcium responses. (**k–n**) Calcium imaging experiments were performed in cultured DRG neurons. Grey traces indicate single cell calcium responses, ratio F(380/F340). Colored line: average of 50 cells. Calcium responses evoked by consecutive addition of 10 µM OxPAPC (), 10 µM AITC (), 1 µM capsaicin (), and 90 mM potassium chloride (), in presence of E06 mAb ( 1/300, **k**,**m**,**n**) or IgM isotype control Ab ( 1/300, **l–n**). (**m**,**n**) Summary data are displayed as percentages of reacting cells and AUC analysis of OxPAPC-induced calcium responses. (**a–n**: OxPAPC: n = 7–13; AITC: n = 5–7; DRGs: n = 7–8, boxplot, median; one-way ANOVA post hoc Holm-Sidak, *p ≤ 0.05).

*In vitro*, stimulation of HEK-293_TRPA1_ with a mixture of OxPAPC and E06 mAb significantly diminished the intracellular increase in calcium. E06 mAb reduced the number of OxPAPC sensitive cells by 75% and reduced the AUC by 85%. IgM isotype control Ab did not change OxPAPC-evoked calcium influx via TRPA1 (Fig. 8a–c,g,h). In contrast, AITC-induced activation of TRPA1 was marginally reduced by E06 mAb and no significant changes in the number of responding cells and the AUC were detected when compared with the IgM isotype control Ab (Fig. 8d–f,i,j).

In mouse DRG neurons, preincubation with E06 mAb, but not the IgM control antibody, reduced the number of OxPAPC responsive neurons by 48%, while the AUC of the remaining reacting neurons was not significantly altered by either treatment (Fig. 8k–n). Similar results were obtained in rat DRG neurons (Supplementary Fig. 7d). E06 mAb did not block AITC or capsaicin-induced DRG activation. Overall, E06 mAb, but not the control IgM antibody, reduced OxPAPC-induced Ca^2+^ influx in HEK-293_TRPA1_ cells and cultured DRG neurons.

### Blockade of OxPAPC-induced TRPA1 activation by ApoA-I mimetic peptide D-4F *in vitro* and *in vivo*

**Figure 9:**
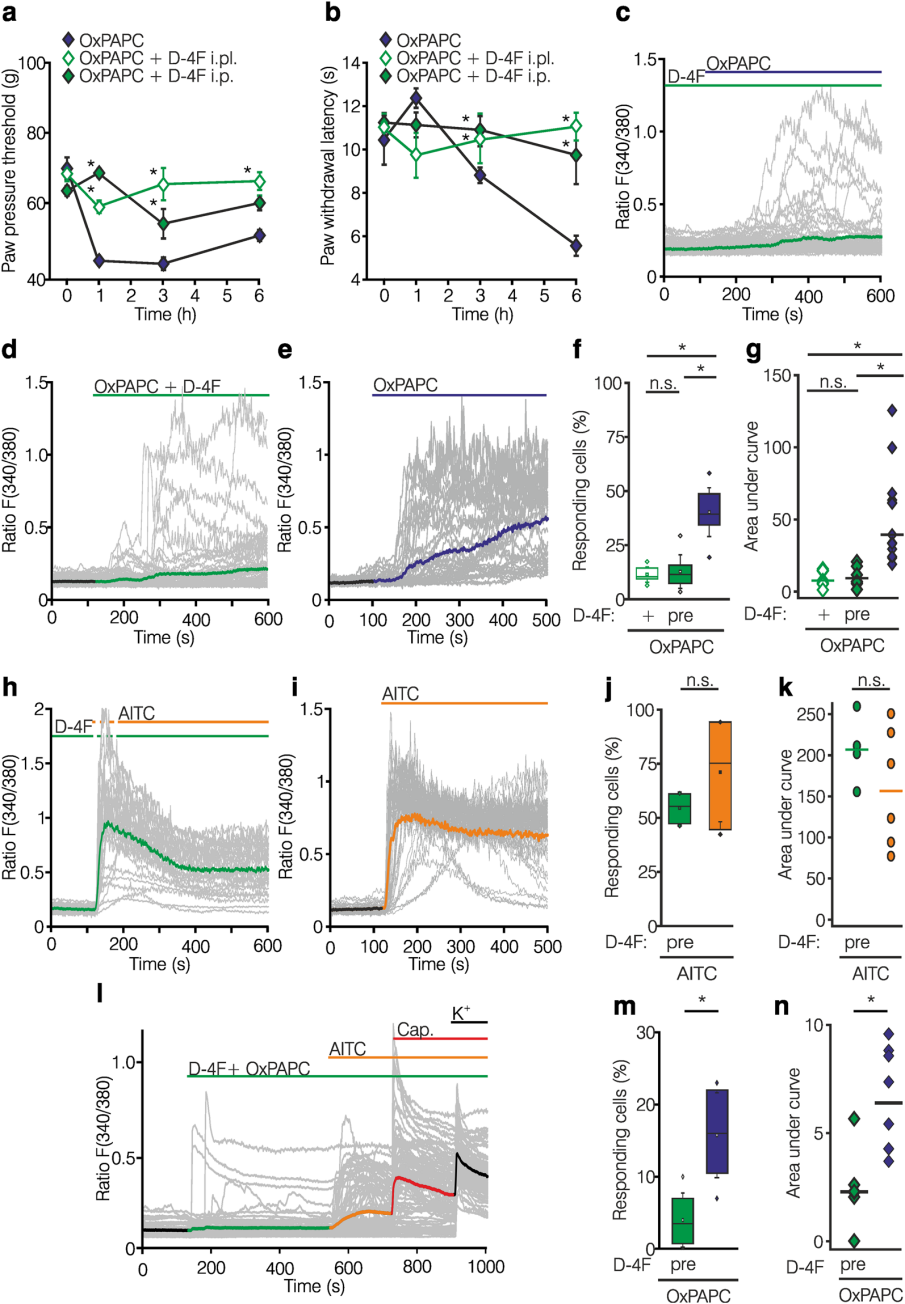
The apo-A1 mimetic peptide D-4F reverses OxPAPC-induced hyperalgesia and TRPA1 activation in HEK-293_TRPA1_ cells and DRG neurons. (**a**,**b**) Pain behavior is shown in Wistar rats treated with intraplantar (i.pl.) OxPAPC alone (, 500 µg), together with D-4F (, 2.5 mg/kg), or after daily injections of D-4F for 5 d (, 12.5 mg/kg intraperitoneal, i.p.) followed by OxPAPC i.pl. Mechanical (paw pressure thresholds, **a**) and thermal (paw withdrawal latencies, **b**) nociceptive thresholds are depicted before and after OxPAPC injection. (**c–k**) Calcium imaging experiments of Fura-2/AM loaded HEK-293_TRPA1_ are depicted after pre-incubation of the cells with D-4F ( 100 µg/ml, 30 min) and subsequently co-application of OxPAPC (10 µM, **c**) or pre-incubation of OxPAPC with D-4F (**d**) for 30 min before application compared to OxPAPC alone ( 10 µM, **e**) and are summarized in (**f**,**g**). (**h–k**) As a negative control, calcium responses in the presence (**h**,  100 µg/ml, 30 min preincubation) or absence of D-4F (**i**) stimulated by AITC ( 10 µM) in HEK-293_TRPA1_ are displayed and analyzed in (**j**,**k**). (**l**,**m**) In mouse DRG neurons, intracellular calcium changes after stimulation with a pre-incubated mixture of D-4F ( 100 µg/ml) and OxPAPC ( 10 µM, **l**) are depicted and summarized (**m**,**n**). (**a**,**b**: n = 3–6 per experiment, mean ± SEM, two-way ANOVA RM post hoc Holm-Sidak, *p ≤ 0.05; (**c**–**m**) HEK-293_TRPA1_: n = 4–13 per experiment; DRGs: n = 6–8 per experiment, boxplot, median, two samples t-test or one-way ANOVA post hoc Holm-Sidak, *p ≤ 0.05).

D-4F, a peptide deduced from the ApoA-I protein, is known to bind OxPAPC and to moderate the development of atherosclerotic plaque lesions^37, 38^. For this reason, we tested whether D-4F could reverse OxPAPC-induced hyperalgesia. In rats, D-4F prevented OxPAPC-induced mechanical and thermal hyperalgesia either after local intraplantar co-injection or following a five day period of systemic intraperitoneal pretreatment (Fig. 9a,b). No alterations in mechanical and thermal nociceptive thresholds were seen following intraplantar injection of D-4F alone (Supplementary Fig. 9a,b) or in contralateral paws (Supplementary Fig. 9c,d).

*In vitro*, pre-treatment of HEK-293_TRPA1_ with D-4F and subsequent stimulation with OxPAPC or with a mixture of OxPAPC and D-4F (preincubated for 30 min), both, lowered the efficiency of OxPAPC to induce TRPA1-mediated calcium influx (Fig. 9c–g). With either treatment, the number of responding cells decreased by 70% and the AUC by 80%. No change in the number of reacting cells or the area under the curve (AUC) was observed when TRPA1-expressing cells were preincubated with D-4F and activated with AITC (Fig. 9h–k).

D-4F also reduced the OxPAPC-induced stimulation of mouse DRG neurons (Fig. 9l–n). Indeed, D-4F significantly diminished the number of responding cells by 64% and the AUC by 62% when DRG neurons were treated with a mixture of OxPAPC and D-4F. No change was seen in AITC-induced Ca^2+^-influx, when D-4F was present. Data were confirmed in rat DRG neurons (Supplementary Fig. 7e).

### D-4F for treatment of inflammatory and arthritis hyperalgesia

**Figure 10:**
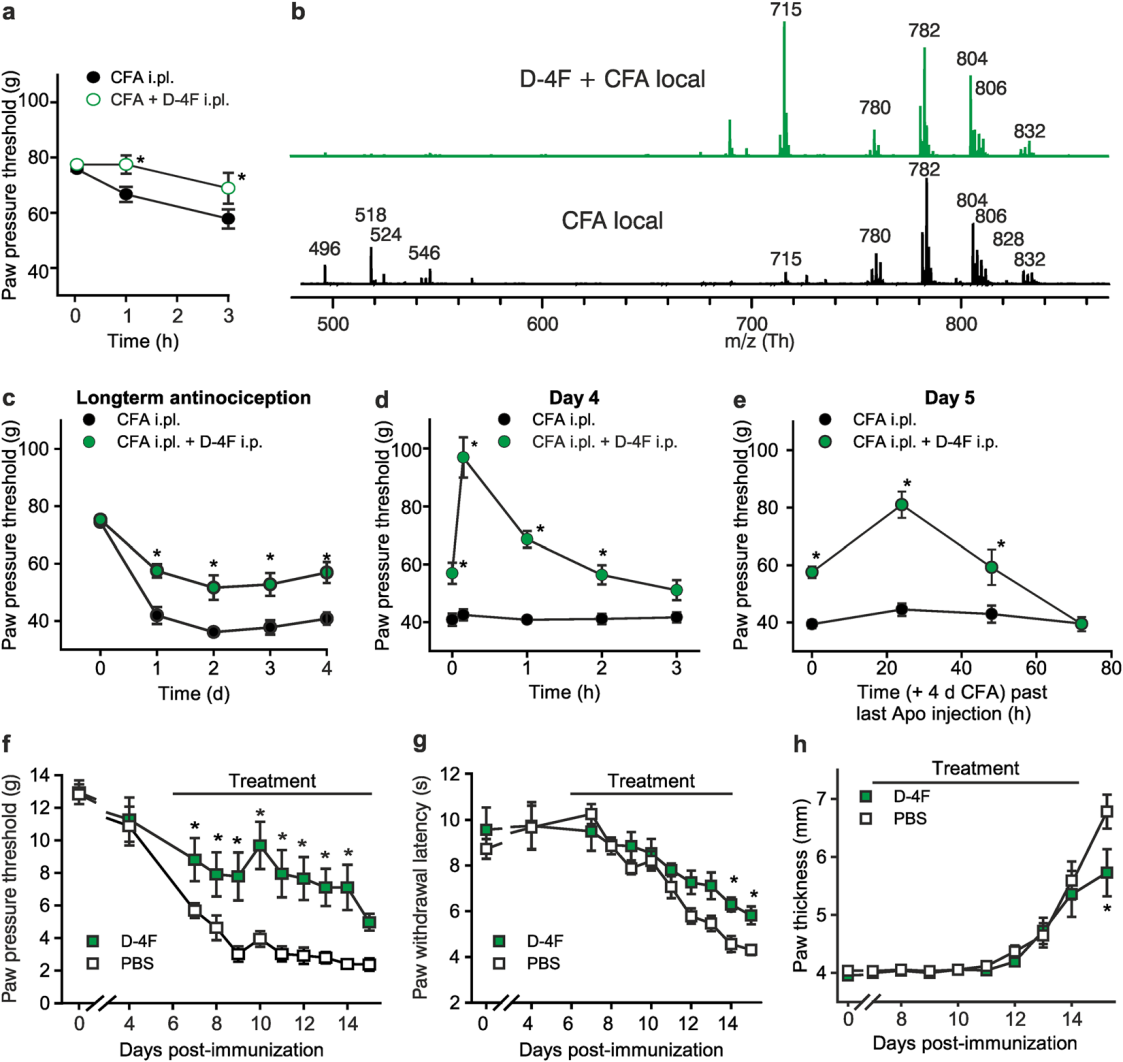
D-4F ameliorates acute and chronic inflammatory hyperalgesia as well as collagen-induced arthritis. (**a**) Mechanical nociceptive thresholds are displayed before and after intraplantar injection of CFA (⚫, 150 µl) or CFA together with D-4F (, 2.5 mg/kg i.pl.) in early inflammation in Wistar rats. (**b**) Analysis of paw tissue by MALDI-TOF MS 3 h after either CFA intraplantarly alone () or CFA together with D-4F treatment depicted () as mass spectra. Oxidized fragments of PAPC were found as LPCs (*m/z* 496.3, 518.3, 524.3 and 546.3) in paw tissue (n = 3, representative example). (**c–e**) Rats with CFA-induced hindpaw inflammation were treated with daily D-4F (12.5 mg/kg/d intraperitoneal, i.p., ) or solvent (•) beginning on day 0. (**c**) Mechanical nociceptive thresholds are shown every day before treatment with D-4F. (**d**) Paw pressure thresholds were measured before and directly after the intraperitoneal D-4F injection for up to 2 h (day 4 shown as an example, day 1–3, Supplementary Fig. 9b–d). (**e**) After the last D-4F injection on day 4 mechanical nociceptive thresholds are presented for up to 72 h. (**f–h**) Collagen-induced arthritis was induced by immunization with collagen in Lewis rats. Rats were treated daily by intraperitoneal injections of D-4F (12.5 mg/kg/d i.p., ), starting at day 6. Mechanical allodynia (**f**) and thermal nociceptive thresholds (**g**) were determined after treatment with D-4F () compared to vehicle treatment (PBS; □). Paw thickness/edema was measured in mm (**h**). n = 8/group, mean ± SEM, two-way ANOVA RM post hoc Holm-Sidak (**a**,**c–e**,**g**,**h**,**f**), *p ≤ 0.05.

The therapeutic potential of D-4F for treatment of inflammatory hyperalgesia was assessed in models of acute and chronic inflammation, meaning CFA-induced hindpaw inflammation^23^ and collagen-induced arthritis^39, 40^. Mechanical hyperalgesia due to CFA was significantly reduced for up to 3 h, when rats were locally treated with a single shot of D-4F (Fig. 10a). No change was observed in thermal hyperalgesia (Supplementary Fig. 9e). In MALDI-TOF MS, PAPC oxidation fragments (i.e. particularly the LPC peak at *m/z* 496.3) were not detectable in paw tissue taken 3 h after injection of CFA plus D-4F compared to CFA alone (Fig. 10b). Peaks of non-oxidized phospholipids (*m/z* about 750–840) remained unaffected.

Repeated daily intraperitoneal injections of D-4F significantly reduced the development of CFA-induced paw edema by 40% from day 2 to day 5 after CFA injection (Supplementary Fig. 10a). Similarly, daily treatments lead to a sustained amelioration of mechanical hyperalgesia when paw pressure thresholds were measured before each daily injection of D-4F (Fig. 10c). On top of this, each daily intraperitoneal D-4F injection completely reversed mechanical hyperalgesia with a maximum effect after 15 min (Fig. 10d, day 1–3: Supplementary Fig. 10b–d). The antinociceptive effect of D-4F persisted for 2 d after the last administration on day 4 after CFA injection (Fig. 10e).

Finally, we assessed whether D-4F was also effective as treatment of chronic inflammatory pain in collagen-induced arthritis (Fig. 10f–h). Immunization of adult female Lewis rats with collagen, emulsified in CFA, induced mechanical hyperalgesia, which developed within one week (Fig. 10f). Thermal hyperalgesia was observed after 10 d (Fig. 10g) as previously described^39, 40^. Hyperalgesia preceded the onset of inflammation as paw thickness (Fig. 10h) and clinical signs of arthritis (Supplementary Fig. 10e) developed 12–14 d after collagen immunization. Daily treatment with D-4F from day 6 to day 15 after immunization, reduced paw edema significantly and clinical scores non-significantly (Fig. 10h and Supplementary Fig. 10e). Importantly, development of both mechanical and thermal hyperalgesia was prevented by D-4F treatment (Fig. 10f,g). Thus, D-4F has potent and long-lasting antinociceptive properties in a preclinical model of rheumatoid arthritis pain and attenuates clinical signs of arthritis.

## Discussion

Our results show experimental evidence that antagonism of the action of OxPL is a potent strategy to target inflammatory pain in rodents. OxPAPC is locally formed in inflamed tissue, and it is a potent pro-nociceptive mediator, which releases the proinflammatory neuropeptide CGRP. OxPAPC species including POVPC and PGPC act predominantly via TRPA1 ion channels, a sensor for chemical stimuli on primary afferent nociceptors. Most relevantly, the pro-nociceptive function of OxPL in acute and chronic inflammation can be effectively blocked either locally by a monoclonal antibody (E06 mAb) or locally and systemically by the ApoA-I mimetic peptide D-4F. These results demonstrate *in vivo* function of OxPAPC as pro-nociceptive mediator and two potent therapeutic strategies, which interfere with OxPAPC-induced pain.

Phospholipids are major constituents of cellular membranes and lipoproteins. During inflammation phagocytes generate ROS^3^. ROS oxidize phospholipids leading to augmented formation of OxPAPC. OxPAPC is a complex mixture of different oxidized phospholipids known to be formed under certain pathophysiological situations such as atherosclerosis, acute lung injury and sepsis as well as neurodegenerative diseases such as Alzheimer’s and Parkinson’s ﻿disease^10, 11^. Here, OxPAPC (100–500 µg $\sim$ 125–625 nmol) was used in a dose comparable to other *in vivo* treatments, e.g. LPS-induced peritonitis^41^ or the air pouch model^42^. Several lipid TRP agonists have previously been used in comparable doses, e.g. hydroxynoneal (150 nmol)^43^, HODE derivatives (100 µg $\sim$ 687 nmol)^22^ or 15d-PGJ_2_ (20 µmol)^44^. *In vitro*, OxPAPC concentrations range from 10 to 100 µg/ml (12.5–125 µmol/ml)^12, 45–48^, which were the basis of our dose selection.

The commercially obtained OxPAPC contained PAPC, POVPC, PGPC and PEIPC. POVPC and PGPC both simulate TRPA1 and likely elicit hyperalgesia. 15d-PGJ_2_, a remote metabolite of OxPAPC, also activates TRPA1. Hence, all of these OxPL species could probably play a role in inflammatory pain. OxPAPC metabolites are not stable for long periods since they rapidly react with other molecules *in vivo*. Typically, some OxPAPC components such as the aldehyde POVPC covalently form Schiff’s bases or Michael adducts with proteins and, therefore, they are only transient pathophysiological mediators^49, 50^. *In vitro* data indicate that enzymes are necessary to metabolize OxPAPC since the composition of OxPAPC in ambient air for up to 5 h barely changes. However, non-oxidized PAPC is highly reactive on ambient air as observed before^12^.

Quantification of the OxPAPC species *in vivo* after injection of either OxPAPC or CFA was accomplished by an E06-based competitive binding assay^51, 52^. We observed higher amounts of E06-immunoreactivity in CFA-inflammation compared to E06-immunoreactivity after local injection of 500 µg OxPAPC (approx. 230 µg/g wet tissue). For comparison, 40–100 µg/g wet tissue OxPAPC species were detected in rabbit aorta^7, 52^ and 5–20 ng/ml POVPC/10–30 ng/ml PGPC in human atheroma plaques^53^. The validity of the competitive binding assay is limited by the specificity of E06 binding to phosphocholine head group of oxidized phospholipids. E06 does not bind to LPC^36^. This suggests that LPC, a hydrolysis end-product of OxPAPC, cannot be the major mediator of the E06-sensitive nociceptive behaviour.

We detected POVPC and PGPC after immediate retrieving tissue by MALDI-TOF while OxPAPC metabolites like LPC were found regularly 15 min after OxPAPC injection. Absolute quantification of individual OxPAPC species by mass spectrometry techniques depends on the use of internal standards for which defined, deuterated oxidized phospholipids would be preferable^54, 55^. Albeit some oxidized phospholipids with a lower reactivity, such as PGPC or POVPC, are available as pure substances and can be used for comparative or semi-quantitative analysis^56, 57^, absolute quantification of our phospholipid samples was technically not yet possible. This was due to the lack of availability of deuterated oxidized phospholipids, or techniques which allow the absolute quantification of individual phospholipid compounds in complex lipid preparations^54, 55^. Rapid metabolism has been observed for other lipids *in vivo* before^58^. In CFA inflammation, OxPAPC species, e.g. POVPC, were endogenously produced in the inflammatory tissue, but peaks differed between animals. Several aspects might explain these findings: (1) increased and continuous ROS production and thereby OxPAPC formation in the course of inflammation, (2) high reactivity of OxPAPC including complex lipid oxidation processes *in vivo* yielding transient products and secondary reactions or rearrangements to generate more stable metabolites (e.g. reaction of the aldehyde with the amino groups of proteins as observed with the TRPA1 protein)^49^ as well as (3) facilitated metabolization of OxPC species by phospholipase A_2_ particularly under inflammatory conditions^59^. Similarly, 15 min after local OxPAPC injection, PGPC and POVPC were not present any more, while LPC was routinely found and decreased in E06 or D4F treated animals. Whether short carbon chain aldehydes and oxidized fatty acids, oxidation fragments of PAPC, also contribute to OxPAPC-induced pain behavior cannot be excluded yet.

OxPAPC was able to induce long-lasting hyperalgesic behavior in rats and mice suggesting that it has not only a fast action as a stimulant of pain behaviour. It might act as a key component in a cascade of proalgesic mediators leading to long-lasting inflammatory pain and sensitization. It was observed that acute OxPAPC-mediated activation of TRP channels was sufficient to release CGRP, thus indicating that OxPAPC triggers elements of the molecular cascade leading to inflammatory pain. Antagonizing OxPAPC function was an efficient way to reduce immediate actions of OxPAPC stimuli such as calcium responses *in vitro* but also long-lasting hyperalgesia *in vivo*.

In nociception, TRPA1 and TRPV1 are of outstanding importance^1^. These ion channels act as detectors of noxious chemical agents on pain-signaling neurons, activating pain pathways to trigger avoidance behavior and even promoting inflammation by release of neuropeptides. Both channels are polymodal receptors and are also sensitive to ROS and peroxidized lipids^21, 22, 60^. For this reason, TRPA1 and TRPV1 are preferential candidate channels for an excitatory action of OxPL on nociceptive neurons.

Five lines of evidence suggest that OxPAPC is an activator of TRPA1: (1) *In vitro* reconstitution of TRPA1 channels in HEK-293 cells shows that OxPAPC and components of OxPAPC such as PGPC and POVPC stimulate TRPA1-mediated ion influx to the cytosol. (2) The TRPA1 blocker HC-030031 inhibits acute OxPAPC-mediated calcium fluxes in DRG neurons. (3) OxPAPC action *in vivo* is antagonized by the TRPA1 blocker HC-030031 and, is attenuated in TRPA1 KO. (4) *In vitro*, OxPAPC-mediated CGRP release is significantly reduced in tissue from TRPA1 KO. (5) OxPAPC dose-dependently oxidizes the TRPA1 peptide, which represents an intracellular N-terminal domain of the ion channel. Recently it was described that exogenous OxPAPC can induce acute pain and persistent mechanical hyperalgesia. *In vivo* application of the TRPA1 inhibitor HC-030031 reversed this effect^33^. This group also showed that OxPAPC can activate TRPA1 *in vitro*, and therefore suggested that targeting the OxPAPC/TRPA1 signaling pathway may be promising in inflammatory pain treatment. This group found no contribution of TRPV1 on OxPAPC function.

Oxidation products of PAPC such as PEIPC share electrophilicity as a common feature with many TRPA1 ligands. Three critical cysteine residues (C621, C641 and C665 in human TRPA1), located in the N-terminal region of the channel, have been proposed to mediate electrophile sensitivity by covalent binding of the ligand and subsequent TRPA1 activation. Recent structural analysis of the TRPA1 channel confirmed the key importance of especially the cysteine residue C621^26^. In calcium imaging experiments, using the hTRPA1 mutant channel lacking the three critical cysteine residues^27, 32^ both, the partly electrophilic mixture of OxPAPC as well as the typical TRPA1 agonist AITC lost their ligand functions, but the channel was still responsive to the non-electrophilic agonist carvacrol. However, the OxPAPC compounds PGPC or POVPV are not electrophilic. This indicates that more than one mechanism leads to OxPAPC-induced activation of TRPA1 and TRPV1. The physiological function of OxPAPC compounds can be direct, e.g. via electrophilic interaction or oxidation of the ion channel, but may also include indirect effects. PGPC and POVPC are known to change the lipid and protein environment of cell plasma membranes^50, 61^ and it might be that the compounds induce mechanical perturbations in the plasma membrane and activate TRPA1 indirectly. This is in accordance with the mechanosensing function of TRPA1^62–64^. Such a mechanism has been proposed for other lipophilic activators of TRPA1 such as bacterial lipopolysaccharides^28^. Furthermore, it would be possible that for instance the carbon acid PGPC, which is known to integrate into the cellular membrane and thought to act only physically with other biomolecules^65^ adds electrostatic charges to the membrane by its polar sn-2 chain. The polar group would be exposed to the aequeous phase^50^. These electrostatic forces might be a trigger for TRPA1 activation^66^. How OxPLs act on TRPV1 is puzzling, but might include electrophilic, cysteine-dependent or cysteine-independent interactions with OxPAPC compounds^67^. Additional experiments will be needed to find out how the single OxPAPC compounds act. Reconstitution of functional TRPA1 or TRPV1 in artificial lipid bilayers and subsequent acute application of OxPAPC oxidation end-products such as PGPC may offer deeper insights in the mechanisms how OxPL interact with membranes and whether this indirect mechanism contributes to the activation of TRPA1 and TRPV1 in nociceptors.

OxPAPC, PGPC and POVPC activate recombinant human TRPA1. OxPAPC induces calcium influx in rat and mouse DRG neurons, and OxPAPC-induced pain behavior was observed in the mouse and rat and was also observed in TRPV1 KO mice. This indicates that TRPA1 activation by OxPL is species-independent, albeit we cannot exclude quantitative differences between species.

In the hTRPA1 peptide binding study, no adduct formation and direct thiol oxidation was detected, suggesting that OxPAPC acts as a two-electron oxidant. Kelch-like ECH-associated protein 1 (Keap1), the oxidative stress sensor, is often activated by the same electrophilic compounds as TRPA1. Keap1 activation by OxPAPC has already been documented and it probably occurs by the same mechanism that we observe here^68^.

Previously, it has been observed that 12.5-fold higher concentrations of OxPAPC (125 µM) induce ROS production in human pulmonary artery endothelial cells while 12.5 µM did not cause this response^69^. Here we show, that 10 µM OxPAPC activates TRPA1 channels. It is unlikely that OxPAPC reacts indirectly through acute ROS production since the activation occurs within seconds. We show that high concentrations of OxPAPC result in an increased oxidizing activity on a peptide mimicking the electrophilic binding site of TRPA1. For these reasons, it is plausible that very high concentration of OxPAPC can also induce ROS production.

Pharmacological TRPA1 and TRPV1 blockade reversed OxPAPC-induced mechanical hyperalgesia, while thermal hyperalgesia was only affected by TRPV1 in rats. In previous studies, TRPA1 and TRPV1 agonists equally triggered CGRP release from mouse skin^70^. In line with this, TRPA1 and TRPV1 antagonists, TRPA1 KO, TRPV1 KO and TRPA1/TRPV1 double KO mice showed reduced OxPAPC-triggered CGRP release to a similar extent. TRPA1 preferentially mediated mechanical hyperalgesia in rats (as shown by the TRPA1 antagonist HC-030031) but mechanical hyperalgesia was only partly reduced in TRPA1 gene deficient mice. Thermal OxPAPC-induced hyperalgesia was completely abolished in TRPV1 deficient mice. Both receptors contributed to OxPAPC-induced CGRP release. In the cellular models, however, TRPA1 channels were the preferential direct targets of OxPAPC. One explanation for this finding could be that the responsiveness of TRPV1 depends on its phosphorylation status, which is raised by inflammatory mediators such as PGE_2_ and bradykinin, and also by TRPA1 activation itself, which stimulates protein kinase A-mediated phosphorylation^71^. TRPV1 activation might be also facilitated by intracellular phosphorylation induced by other OxPAPC-binding receptors (CD36, PAF receptor, toll like receptor 4, peroxisome proliferator-activated receptors, prostaglandin E2 receptor)^48^ on TRPV1 expressing neurons. It needs to be considered that *in vivo* inflammatory mediators are abundant in the CFA-injected paw. OxPAPC could induce Toll-like receptor 4 activation possibly in CCR2-expressing monocytes or macrophages, which then mediate indirect, sensitizing actions on TRPV1 function^9, 42, 72^. Furthermore, TRPA1 function is increased via TRPV1-induced Ca^2+^-influx^73^. In summary, our data suggest that *in vivo* the interaction of TRPA1 and TRPV1 is necessary for OxPAPC action.

Besides pro-inflammatory functions, OxPAPC has anti-inflammatory purposes. Recently, it was shown that the prostanoid-like OxPL epoxycyclopentones are the potent anti-inflammatory OxPL activating transcription factor nuclear factor E2-releated factor 2 (nrf2)^12^. OxPL species with high anti-inflammatory properties include PEIPC or PECPC, but not POVPC or PGPC. OxPAPC has several known receptors including CD36, PAF receptor, toll like receptor 4, peroxisome proliferator-activated receptors, prostaglandin E2 receptor^48^ or VEGF receptors^7^. Since none of these are expressed in HEK-293 cells we think that this mechanism in not relevant *in vitro*, however, we cannot completely rule out that *in vivo* other mechanisms play a role besides TRPA1. Apparently, the net outcome of pro- or anti-inflammatory effects depends on the biological context.

We identified two strategies to treat inflammatory hyperalgesia by blocking OxPAPC function. Strategy 1 involved anti-OxPAPC antibodies. OxPL are immunogenic and can be regarded as “neo”-self antigens^7^. OxPLs can initiate both innate and adaptive immune responses. This is of major relevance in atherosclerosis^7^. Autoantibodies to epitopes of OxLDL are highly abundant in the plasma of humans and other mammals^36^. These autoantibodies recognize epitopes of OxLDL in atherosclerotic lesions where they can form immune complexes with OxLDL^74^. In a key study by Palinski *et al*.^18^, multiple monoclonal antibodies (called E0 antibodies) against various epitopes of OxLDL were isolated from apoE-deficient mice. One of these monoclonal antibodies, the E06 mAb, attracted considerable attention, because it recognizes a variety of OxPL metabolites and is therefore useful in diagnostics of atherosclerosis and exhibits promising therapeutic properties^75^. Indeed, the phosphorylcholine head group is essential for E06 binding, but its binding also depends on the oxidized phospholipid conformation including fatty acyl chain length^36, 76^. In our study, we now show that E06 mAb has the potential to reduce hyperalgesic behavior, most likely because it acts as a function-blocking antibody against OxPAPC, which is formed in inflammatory tissue. When E06 is pre-incubated with OxPAPC the agonist function of OxPAPC on TRPA1 channel activation is reduced, indicating that E06 is a potent antagonist to functional OxPAPC metabolites. This suggests that humanized monoclonal anti-OxPAPC antibodies might be novel therapeutics for the treatment of inflammatory pain. Since anti-OxPL antibodies naturally rise in the plasma of humans^74^, it can be assumed that therapeutic antibodies against OxPAPC are well tolerated if given locally at the site of inflammation.

Strategy 2 used D-4F peptide to reduce inflammatory hyperalgesia. In recent years, ApoA-I mimetic peptides have received high attention as potential therapeutic agents, because these synthetic peptides demonstrated anti-inflammatory and anti-atherosclerotic activity *in vivo* ^77^. Furthermore, ApoA-I mimetic peptides are just emerging for the treatment of autoimmune disease, endotoxemia and cancer^77^. Among the best-studied ApoA-I peptides is D-4F, which is already tested in clinical phase II studies^78, 79^ and is well tolerated by patients^80^. D-4F is synthesized from D-amino acids and shows a remarkably high affinity to OxPL^37^. For instance, D-4F and its homolog L-4F bind pro-inflammatory OxPL at low nanomolar concentrations. The binding affinity of this interaction is orders of magnitude higher than the affinity of OxPL to human ApoA-I, the natural binding partner^37^. Apo-mimetic peptides, however, are not specific for OxPAPC, but the affinity of D-4F to PGCP, POVPC, PEIPC and 1-(palmitoyl)-2-(5-keto-6-octene-dioyl) phosphatidylcholine (KOdiaA-PC) is substantially higher than for PAPC or other nonoxidized lipids, cholesterol and oxidized lipids like 5(S)-hydroperoxy-5Z,8Z,10E,14Z-eicosatetraenoic acid (HPETE)^37^. In this study, we tested whether systemic administration of D-4F has an antinociceptive effect in animal models of acute and chronic inflammation. In collagen-induced arthritis in rats, a model for chronic inflammation, intraperitoneal injections of D-4F raised mechanical and thermal nociceptive thresholds. In acute CFA-induced inflammation, D-4F treatment showed long-term reduction of mechanical hyperalgesia. Mechanistically we suggest that the antinociceptive property of D-4F is due to a scavenger-like inhibition of OxPAPC function, thus antagonizing the pro-inflammatory and pro-nociceptive properties of OxPAPC metabolites. This proposal is based on the following results: D-4F (1) reduces the abundance of OxPAPC fragments, (2) inhibits OxPAPC-induced hyperalgesic behavior, and (3) interferes with OxPAPC ligand function on TRPA1, and to a lesser degree TRPV1 ion channels (heterologously expressed in HEK cells and native TRPA1 in rat and mice DRG neurons).

In conclusion, we provide experimental evidence that OxPL are activators of TRPA1 and TRPV1 *in vitro* and *in vivo* and that antagonism of OxPL and specifically OxPAPC is beneficial for the treatment of acute and chronic inflammatory pain. Potential pharmacotherapies can either be based on monoclonal antibodies with E06-like properties, or ApoA-I mimetic peptides, such as D-4F.

## Materials and Methods

### Reagents and Chemicals

The following reagents were used: OxPAPC and PAPC (Hycultech GmbH, Beutelsbach, Germany), AITC, carvacrol, capsaicin, HC-030031, BCTC, 2,5-dihydroxybenzoic acid (0.5 M in methanol), GSK2193874 (Sigma-Aldrich, Taufkirchen, Germany), 2-Aminoethoxydiphenylborane (2-APB; Merck, Darmstadt, Germany), E06 mAb (Avanti Polar Lipids, Alabaster, AL, USA), mouse IgM isotype control (Biomol GmbH, Hamburg, Germany), D-4F peptide (Peptide Specialty Laboratories GmbH, Heidelberg, Germany), CFA (Calbiochem, San Diego, USA/BD Bioscience, San Jose, USA). Dimethyl sulfoxide or aqueous physiological solutions served as solvents. Standard chemicals and reagents are given in the supplementary information.

Chemicals and reagents for cell culture experiments included gentamycin, hygromycin, nerve growth factor (NGF), IST liquid media supplement (100x), HEPES (4-(2-hydroxyethyl)piperazine-1-ethanesulfonic acid, N-(2-Hydroxyethyl)piperazine-N′-(2-ethanesulfonic acid)), NMDG (N-methyl-d-glucamine), poly-L-lysine, Dulbecco’s minimum essential medium (DMEM; Sigma-Aldrich, Taufkirchen, Germany), DMEM/F12, trypsin, fetal bovine serum, penicillin, streptomycin, Lipofectamin 2000 (Life Technologies GmbH, Darmstadt, Germany), Earle’s minimum essential medium (MEM; Biochrom, Berlin, Germany), geneticindisulfic acid (G418; Carl Roth, Karlsruhe, Germany); bovine type II collagen (MD Bioproducts, Egg, Switzerland). Reagents for *in vivo* experiments included isoflurane (cp pharma, Burgdorf, Germany/Abbott, Maidenhead, UK) and embutramide (T61; Intervet GmbH, Unterschleissheim, Germany).

### Animals

Male Wistar rats (Janvier, Saint-Berthevin Cedex, France) and female Lewis rats (Charles River, Margate, Kent, UK) were used in the experiments. For injection of agents to rats, a brief isoflurane anesthesia was performed. Rats were euthanized in deep isoflurane anesthesia by intracardiac injection of a solution of T61 (embutramide, mebezonium and tetracaine) or an overdose of pentobarbital.

C57BL/6 J wild type mice were bred in Institute of Clinical Neurobiology, University of Wuerzburg, Germany. Initial TRPA1 KO mice were a generous gift from Drs Kwan and Corey^63^ and TRPV1 KO mice were donated by Dr. Davis^81^. All mutant mice were bred in the Institute of Physiology and Pathophysiology, University of Erlangen, Germany. Mice were asphyxiated in CO_2_.

All animals were kept under a nonsterile housing environment in accordance with the local Animal Care and Use Guidelines. Animals were randomly assigned to treatment groups.

### Lipid extraction and MALDI-TOF MS

Paw tissue from treated animals (see below) was shock-frozen after dissection from bone and skin followed by homogenization in a mixture of chloroform, methanol and water (2:1:1 volume) with a lab mixer (IKA® RW 14, IKW®-Werke, Staufen, Germany). After centrifugation (1500 g, 4 °C, 30 min), the chloroform (lower) phase was recovered, evaporated under nitrogen and stored at −20 °C. MALDI-TOF MS was performed immediately after extraction using 2,5-dihydroxybenzoic acid as matrix, as described earlier^82^. Briefly, chloroform extracts or reagents (OxPAPC, PAPC; CFA)^83^ were re-dissolved in 20–30 µl of a 2,5-dihydroxybenzoic acid matrix solution (in methanol) prior to deposition onto the MALDI target. MALDI-TOF mass spectra were acquired on a Bruker Autoflex mass spectrometer, which is equipped with a pulsed nitrogen laser, emitting at 337 nm (Bruker Daltonics, Bremen, Germany). The extraction voltage was 20 kV. Gated matrix suppression was applied to prevent the saturation of the detector by matrix ions. For each mass spectrum, 128 single laser shots were averaged. The laser fluence was kept about 10% above threshold (i.e. the minimum laser fluence required to achieve detectable signals) to obtain optimum signal-to-noise (S/N) ratios. In order to enhance the spectral resolution all spectra were acquired in the reflector mode using delayed extraction conditions.

### E06 competitive binding assay

The competitive binding assay was designed according to Cox *et al*.^84^. OxPAPC-coated Snooper (Avanti Polar Lipids, Alabaster, AL, USA) were blocked with BSA (2%) and 0.27 mM EDTA in PBS for 45 min. Meanwhile, lipid extracts as well as OxPAPC in different concentrations as a standard curve was emulsified in PBS and mixed with 2 µg/ml TopFluor-labeled E06 mAb dissolved in 1% BSA/PBS containing 0.135 mM EDTA^51, 52^. After 4x washing the snoopers with PBS, the mixture of lipid plus antibody was added and incubated for 1 h at room temperature. Wells were washed 3 times with PBS. Fluorescence intensity was measured in distilled water at 485 nm with a Tecan Genios pro Fluorescence plate reader (Tecan group ltd., Männedorf, Switzerland). Concentrations were calculated from a standard curve obtained with OxPAPC (0.006–20 µg).

### hTRPA1 peptide binding of OxPAPC

A model peptide of the intracellular N-terminal part of hTRPA1 including amino acids 607–670 (UniProt database, O75762, Thermo Fischer scientific) was used to evaluate the effect of OxPAPC on cysteine residues of TRPA1^30^. The peptide (50 M) was dissolved in 20 mM ammonium bicarbonate buffer, pH 7.4, and treated with 50 and 500 M OxPAPC for 15 min. The samples were injected using a syringe pump at a flow rate of 240 mLh^−1^ into a maXis 4G, a ultra-high-resolution ESI-TOF mass spectrometer (Bruker, Bremen, Germany). Nitrogen was used as the nebulizing gas at a pressure of 10 psi and as the drying gas at a temperature of 180 °C and a flow rate of 5 L min^−1^. All experiments were carried out in the positive-ion mode and obtained spectra deconvoluted and further processed in a data analysis software provided by Bruker Daltonics. Analytical samples were prepared by mixing peptide samples in water with a mixture of 0.1% formic acid in water/acetonitrile (1:1, v/v).

### CGRP release

CGRP release was measured from skin flaps from both hindpaws as described earlier^85^. Samples were directly placed in test tubes, washed for 30 min in carbogene gassed synthetic interstitial fluid (SIF) containing (in mM) 108 NaCl, 3.48 KCl, 3.5 MgSO_4_, 26 NaHCO_3_, 1.7 NaH_2_PO_4_, 1.5 CaCl_2_, 9.6 sodium gluconate, 5.5 glucose und 7.6 sucrose. CGRP release was measured in a shaking bath at 37 °C basally and several incubation steps designed in an intraindividual application scheme. Chemical stimulation with OxPAPC (30 µM) with/without antagonists occurred in a third incubation step and was followed by two incubations in SIF. To block TRPV1 and TRPA1 receptors, respectively, antagonists were added from the second to the fourth incubation period. CGRP was quantified by an enzyme immunoassay kit (Bertin Pharma, Montigny le Bretonneux, France) and read out photometrically (Dynatech, Channel Islands, UK). The antibodies used are directed against human α/β-CGRP but are cross-reactive against mouse CGRP.

### Cell culture and cell lines

Untransfected HEK-293 cells were maintained in DMEM, 4.5 g glucose, 1 mM L-glutamine, 10% FCS, 1% penicillin/streptomycin. For HEK-293 cells stably expressing rTRPV1-YFP, medium was supplemented with geneticin (0.5 mg/ml). HEK-293 cells expressing TRPV2, TRPV3, and TRPV4 were described earlier^34^.

### Stable cell line HEK-293_hTRPA1_

Human TRPA1 cDNA was cloned to the lentiviral expression vector pCDH1-UBC-MCS-EF1-Hygromycin clone 1 (SBI, Mountain View, CA, USA). Lentiviral particles were produced in HEK-293T cells with the pseudotyping vector pMD2.G and the packaging vector pCMVΔR8.91. TRPA1 positive clones were selected by hygromycin (0.4 mg/ml) and the stable cell line was continuously kept under hygromycin selection.

### DRG preparation

Preparation of DRG from adult wildtype mice and adult rats were carried out as described^34^. DRG neurons were grown at a density of 7 × 10^3^ cells per glass cover slip coated with poly-L-lysine (20 µg/ml) and cultured at 37 °C, 5% CO_2_ atmosphere for one day. Measurements were performed the following day. Medium contained 100 ng/ml nerve growth factor (Sigma-Aldrich, Taufkirchen, Germany).

### Calcium imaging

For ratiometric single cell calcium analysis, either DRG neurons or HEK-293 cells were labeled with 6 µM Fura-2/AM for 30 min at 37 °C in imaging solution (in mM): 134 NaCl, 6 KCl, 1 MgCl_2_, 1 CaCl_2_, 10 HEPES, 5.5 glucose, pH 7.4 adjusted with NaOH^86^. All measurements were performed at room temperature using a Nikon TE2000-E microscope. Fura-2/AM was excited for 100 ms with 340/380 nm with a Lambda DG4/17 wavelength switch (Sutter Instruments, Novato, CA, USA). Time-lapse image series were acquired at intervals of 2 s with a cooled EMCCD Andor iXon camera (Andor Technology Ltd., Belfast, UK) controlled by NIS Elements Software (Nikon, Düsseldorf, Germany). Objective: CFI S-Fluor 10x/0.5 (Nikon). Image series were analyzed with ImageJ 1.46r, time series analyzer V2.0 plugin (Rasband, W.S., ImageJ, U. S. National Institutes of Health, Bethesda, Maryland, USA). The following modulators were used: OxPAPC, AITC, capsaicin, carvacrol, BCTC, HC-030031, E06 mAb, IgM isotype control Ab, ApoA-I mimetic peptide D-4F.

In all statistical analysis, the mean of basal fluorescence intensity was determined for each measurement. Number of reacting cells (%) was calculated by 1.5-fold increase of mean basal fluorescent intensity after stimulation. The area under curve (AUC) was taken from the mean. Intervals correspond to the stimulation period of each substance. Mean of basal fluorescence was set as zero.

### Whole cell patch clamp recordings

HEK-293 cells (10^5^) cells were placed in a recording chamber. Pipettes with 2.5–5 MΩ resistances were pulled from borosilicate glass (GB 150-8P, Science Products, Hofheim, Germany) with a P-97 micropipette puller (Sutter Instruments, Novato, CA, USA). Whole cell patch clamp measurements were performed with HEKA EPC-10 USB and controlled by patch master software (HEKA Electronic, Lambrecht, Germany)^86^. Ramps from −100 to +100 mV of 500 ms duration were sampled continuously at 5 kHz every second. Currents were filtered at 2.9 kHz. When indicated, sodium chloride was partly replaced by N-methyl-D-glucamine (75 mM) to reduce the contribution of sodium ions to inward currents^35^. For measurements of currents at constant voltage, cells were clamped at −60 mV in whole cell configuration. Agonists were applied with a second pipette controlled by a pneumatic drug ejection system (0.5 s, 0.4 bar; PDES-02DX, npi electronic, Tamm, Germany). Extracellular solution (in mM): 145 NaCl, 5 CsCl, 2 MgCl_2_, 10 HEPES, 10 glucose, pH 7.4 (HCl). Intracellular solution (in mM): 140 CsCl, 4 MgCl_2_, 10 HEPES, 10 EGTA, pH 7.2 (CsOH). For measurements shown in Supplementary Fig. 5b, pentasodium tripolyphosphate hexahydrate was added to the intracellular solution^87^.

### Hindpaw inflammation and pain behavior testing

Randomly assigned male Wistar rats received intraplantar injections of either 150 µl CFA^23^ or 100–500 µg OxPAPC/PAPC/paw (125–625 nmol dissolved in 100 µl 0.9% saline) under isoflurane anesthesia. The doses were chosen in accordance with previous publications of OxPAPC injection or applications of other oxidized lipids^22, 42, 43^. In selected experiments rats were treated with inhibitors as described in the legends either by local intraplantar or systemic (intravenous or intraperitoneal) injection: BCTC (4 mg/kg i.v.), HC-030031 (30 mg/kg i.p.), E06 mAb (1/100: 0.01 µg/paw), D-4F (2.5 mg/kg i.pl.: 500 µg/paw or 12.5 mg/kg i.p.). In TRPA1 and TRPV1 KO mice, 100 µg of OxPAPC was suspended in 25 µl 0.9% saline and injected i.pl. Mechanical nociceptive thresholds in rats were determined using the paw pressure algesiometer (modified Randall-Selitto test; Ugo Basile, Comerio, Italy) as described^88^. The pressure required to elicit paw withdrawal, the paw pressure threshold, was determined in rats. In mice, mechanical allodynia was measured by the up- and down method using von Frey hair filaments^40^. First filament used was number five. Vigorous paw withdrawal or licking was considered as a positive trial. Each filament was applied at least three times. When two out of three trials were positive the response was considered positive, the next filament with the higher number was applied. In case of a negative response, the next filament with the lower number was used. The sequence was continued for four more responses after the first positive response. The resulting pattern of the responses was used to calculate the 50% threshold using the following formula: 50% g threshold = (10^[*X*^_f_^+kδ]^)/10,000. (X_f_ = value of the last applied filament; κ = tabular value of the pattern of the responses and δ = mean difference between the filaments).

Thermal nociceptive thresholds for rats and mice were obtained by measuring the paw withdrawal latency according to Hargreaves using the plantar test apparatus (Ugo Basile) as previously described^3^. Here, rats were habituated for 15 min to individual plexiglas chambers placed on a glass floor at room temperature. A beam of radiant heat was applied onto the plantar surface of each hindpaw and paw withdrawal latencies (s) were recorded. Withdrawal latencies were monitored three times in each paw alternately and the latencies of both paws were average on each measurement day. At least one minute was given between consecutive measurements in the same paw and a cut-off latency time of 20 sec was used to avoid tissue damage. Averages from three measurements per treatment were calculated. Baseline measurements were obtained before agent injections and at the indicated time points thereafter. Paw edema was determined by placing hindpaws in a water-filled Perspex cell of a plethysmometer (Ugo Basile, Comerio, Italy).

### Collagen-induced arthritis and pain behavior testing

Induction of arthritis was performed as described previously^40^. Briefly, bovine type II collagen (4 mg/ml; MD Bioproducts, Zürich, Switzerland) was dissolved in acetic acid (0.1 M) and then emulsified with CFA (1 mg/ml). Under isoflurane anesthesia female Lewis rats were injected intradermally at the base of the tail with 200 µl of the collagen (400 µg)/CFA emulsion. The onset of arthritis scores was around day 12 (see Fig. 10f–h). Rats were scored on a scale of 0–3 per hindpaw; 0–6 per rat. Clinical signs of arthritis and body weight were monitored prior to immunization and then during the disease process. Mechanical hindpaw withdrawal thresholds were assessed by applying a series of calibrated von Frey filaments (0.4–15.0 g; North Coast Medical, Gilroy, CA, USA) to the plantar surface of the hindpaw according to the “up-down” method. Heat hyperalgesia was assessed as described above. Paw swelling was determined by measuring the thickness of each hindpaw using a thickness gauge (Mitutoyo, Leonberg, Germany; expressed in mm).

### Statistics

Data are presented as mean SD/S.E.M., median and boxplot, defined as followed: box = upper/lower quartile, whisker = 1x SD, line = median, square = mean, diamonds = upper/lower outliner. Multiple measurements with one or two variables were analyzed by one-way ANOVA or by two-way ANOVA repeated measurements (RM), post hoc test as indicated. Differences were considered significant when p ≤ 0.05. Statistical analysis for *in vitro* data was performed with OriginPro 9.0 (OriginLab Corporation, Northampton, MA, USA) and behavior studies with SigmaStat (Systat Software GmbH).

### Study approval

All experiments and study protocols were performed in accordance with the European Union guidelines, and were approved by our institutional Animal Care and Utilization Committee (Regierung von Unterfranken, Wuerzburg, Germany) and by the Home Office, London, UK, and in accordance with the International Association for the Study of Pain.

## Electronic supplementary material

Supplementary data
