# Supplementary material for: Inflammatory pain control by blocking oxidized phospholipid-mediated TRP channel activation
Source: Sci Rep. 2017 Jul 14;7:5447. doi: 10.1038/s41598-017-05348-3 (PMC5511297; doi:10.1038/s41598-017-05348-3)
Supplement: Supplementary file 1 — Supplementary data [file 41598_2017_5348_MOESM1_ESM.doc]

## Inflammatory pain control by blocking oxidized phospholipid-mediated TRP channel activation

## Beatrice Oehler^1,2,8^, Katrin Kistner^3,8^, Corinna Martin^1^,^2^, Jürgen Schiller^4^, Rafaela Mayer^1,2^, Milad Mohammadi^1,2^, Reine-Solange Sauer^1^, Milos R. Filipovic^5^, Francisco R. Nieto^6^, Jan Kloka^1,2^, Diana Pflücke^1^, Kerstin Hill^7^, Michael Schaefer^7^, Marzia Malcangio^6^, Peter W. Reeh^3^, Alexander Brack^1,8^, Robert Blum^2,8^, Heike L. Rittner^1,8^

^1^ Department of Anesthesiology, University Hospital of Wuerzburg, Wuerzburg, Germany
^2^ Institute of Clinical Neurobiology, University Hospital of Wuerzburg, Wuerzburg, Germany
^3^ Institute for Physiology & Pathophysiology, University of Erlangen-Nuremberg, Erlangen, Germany
^4^ Institute for Medical Physics and Biophysics, University of Leipzig, Leipzig, Germany
^5^ Department of Chemistry and Pharmacy, Friedrich-Alexander University Erlangen-Nuremberg, Germany
^6^Wolfson CARD, King's College London, Guys' Campus, London, United Kingdom
^7^ Rudolf-Boehm-Institute for Pharmacology and Toxicology, University of Leipzig, Leipzig, Germany

**Supplementary Material and Methods**

Superoxide detection with dihydroethidium

For analysis of ROS production induced by OxPAPC, 100.000 HEK-293_TRPA1_ cells grown for 24 h on coated glass coverslips were loaded with 10 µM dihydroethidium (Sigma-Aldrich, Taufkirchen, Germany) for 15 min at 37°C. Cells were treated with either carbonyl cyanide-*4*-(trifluoromethoxy)phenylhydrazone (FCCP, 10 µM), H_2_O_2_ (100 µM), or OxPAPC (10 µM) in calcium imaging buffer (as described above) and were analysed after incubation for 10, 20 and 30 min at 37°C. As control, cells were incubated in calcium imaging buffer only. DHE labels were analysed with life cell imaging in calcium imaging buffer. For that purpose, cells were excited with a LED light source (550 nm, CoolLED) and fluorescence (emission: 574 – 650 nm) was imaged with a CCD-camera (Rolera XR, Qimaging), mounted on an Olympus BX51WI microscope with an Olympus objective (40x water, N.A. 0.8). 20 images (8 bit) were acquired at 10 Hz, at indicated time points. Images were averaged and integrated fluorescence intensity was determined in regions of interest representing individual cells. For image analysis the software ImageJ was used.

**Supplementary Tables**

| **Lipid species** | **Peak Position (*m/z*)** | **Assignment of Molecular Mass** |
| --- | --- | --- |
| Oxidized PAPC  (LPC) | 496 | LPC 16:0 (H^+^) |
|  | 518 | LPC 16:0 (Na^+^) |
|  | 524 | LPC 18:0 (H^+^) |
|  | 546 | LPC 18:0 (Na^+^) |
|  | 551 | Trimer of 2,5-Dihydroxybenzoic acid - 3H^+^ + 4Na^+^ (Gas phase) |
| Fragments of triacylglycerol | 575 | Fragment of triacylglycerol |
|  | 579 | Fragment of triacylglycerol |
|  | 594 | Aldehyde derived from PAPC (*); POVPC |
|  | 610 | Carboxylic acid derived from PAPC (*); PGPC (H^+^) |
| Fragments of triacylglycerol | 601 | Fragment of triacylglycerol |
|  | 616 | Aldehyde derived from PAPC (*); POVPC |
|  | 620 | Ceramide-1-phosphate d18:0/16:0 (H^+^) |
|  | 632 | Carboxylic acid derived from PAPC (*); PGPC (Na^+^) |
|  | 648 | Cerarmide 18:1/24:1 (H^+^) |
| Fragments of triacylglycerol | 689 | Fragment derived from *m/z* 953.8 |
|  | 715 | Fragment derived from *m/z* 979.8 |
|  | 725 | Sphingomyelin 16:0 (Na^+^) |
| PC species | 734 | PC 16:0/16:0 (H^+^) |
|  | 756 | PC 16:0/16:0 (Na^+^) |
|  | 758 | PC 16:0/18:2 (H^+^) |
|  | 760 | PC 16:0/18:1 (H^+^) |
|  | 780 | PC 16:0/18:2 (Na^+^) |
|  | 782 | PC 16:0/18:1 (Na^+^) or PC 16:0/20:4 (H^+^);  unoxidized PAPC |
|  | 804 | PC 16:0/20:4 (Na^+^) |
|  | 806 | PC 16:0/22:6 (H^+^); PEIPC |
|  | 810 | PC 18:0/20:4 (H^+^); PECPC |
|  | 822 | Triacylglycerol 48:1 |
| PC species | 828 | PC 16:0/22:6 (Na^+^); PEIPC |
|  | 832 | PC 18:0/20:4 (Na^+^); PECPC |
| Hydroxylated triacylglycerols | 953 | Triacylglycerol (1×18:2-OH, 2×18:1-OH) (Na^+^) |
|  | 979 | Triacylglycerol (2×18:0-OH, 1×18:0-(OH)_2_) (Na^+^) |

**Supplementary Table 1: *Summary and assignments of (phospho)lipids detected by positive ion MALDI-TOF mass spectrometry.[[1]](#footnote-2)*** Integer m/z values are given.

**Supplementary Figures**

**Supplementary Figure 1: Intraplantar injection of OxPAPC does not alter mechanical and thermal nociceptive thresholds in contralateral hind paws of the same animal. (a, c)** Mechanical (paw pressure thresholds) and (**b, d**) thermal (paw withdrawal latency) nociceptive thresholds were measured on the contralateral hindpaws after ipsilateral intraplantar injection of OxPAPC (100 µg ◆, 200 µg ◆, and 500 µg ◆, n = 6, **a, b**) or PAPC (500 µg ◼, **c, d**) at indicated time points. Mean ± SEM of n = 6/group, two-way ANOVA post hoc Holm-Sidak, p ≥ 0.05.

**Supplementary Figure 2: MALDI-TOF MS of OxPAPC, PAPC, and CFA preparations used for intraplantar injection.** Positive ion MALDI-TOF mass spectra of (**a, b**) commercially available OxPAPC, PAPC exposed to ambient air for up to 5 d and (**c**) complete Freud’s adjuvant (CFA) recorded in the presence of 2,5-dihydroxybenzoic acid as matrix. All peaks are marked by their *m/z* values. The majority of peaks represent aldehydes (*m/z* 594.5 = POVPC) or carboxylic acids (*m/z* 610.5 = PGPC). In OxPAPC samples only small amounts of higher mass oxidation products of PAPC (m/z 828.6 = PEIPC) were detected which disappear within 3 d. In contrast, PAPC was strongly oxidized in ambient air for up to 5 d: first, oxidation products with higher molecular weights (in particular *m/z* 828.6) appeared, which were then degraded into the aldehyde, the carboxylic acid and lysophosphatidylcholine (LPC). After 5 d the amount of LPC decreased again, possibly indicating a further degradation to even smaller (water-soluble) products. Due to the lack of standards of oxidized lipids[^1^](#_ENREF_1) we compared the intensities of selected peaks after *in vitro* air expose of commercial OxPAPC for 5 d. The amount of OxPAPC was less than 10% of the initial value. The amount of POVPC decreased for about 50% only. This indicates that the stability of the observed peaks *in vitro* is as follows: PEIPC < POVPC < PGPC. Peaks representing CFA (**c**) correspond to paraffin oil and mannide monooleate. Both substances are major constituents of the CFA preparation (supplier information, Calbiochem San Diego, USA/BD Bioscience, San Jose, USA).

**Supplementary Figure 3: Thermal and mechanical nociceptive thresholds in contralateral paws are unaltered by TRPA1/TRPV1 inhibitors in rats and in TRPA1 or TRPV1 KO mice.** (**a,c**) Paw pressure thresholds and (**b, d**) paw withdrawal latencies were analyzed in the contralateral paw after co-injection of 500 µg OxPAPC (i.pl. ipsilaterally) plus the TRPA1 channel blocker HC-030031 (**a, b**, i.p. ▼) or the TRPV1 channel blocker BCTC (**c, d**, i.v. ▲) at indicated time points. Mechanical (**e**) and thermal (**f**) thresholds remain unchanged after injection of 100 µg OxPAPC in contralateral paws in TRPA1()- and TRPV1()-KO mice as well as in wild type littermates () (all mean ± SEM, p ≥ 0.05, **e**: n = 6, Kruskal-Wallis ANOVA post hoc Student-Newman-Keuls; **f**: n = 5-6, one-way ANOVA RM post hoc Holm-Sidak, * p ≤ 0.05;).

**Supplementary Figure 4:** **A higher dose of OxPAPC did not increase CGRP release.** CGRP release (pg/ml) of mice paw skin evoked by 100 µM OxPAPC (▌,◆) at indicated time point. Mean ± SEM, n = 8.

**Supplementary Figure 5: Untransfected HEK-293 cells show no OxPAPC-induced calcium response. a** Fura-2 based calcium imaging of untransfected HEK-293 cells upon stimulation with 10 µM OxPAPC (▬). Grey trace depicts single cell calcium levels; the blue line represents the mean value of 100 single cell traces. Experiment represents one of three independent measurements. Ratio F(340/380): relative increase in fluorescence intensity (340/380 nm) representing calcium-induced changes in the Fura-2 ratio. **b** Recording of TRPA1 currents upon stimulation with AITC, OxPAPC, or PGPC (each 10 µM) in presence of polyphosphate (10 mM) in the intracellular buffer solution measured at -60 mV voltage clamp.

**Supplementary Figure 6: No significant influence of antioxidants on and no ROS production by OxPAPC in HEK-293_TRPA1_.** (**a, b**) Summary of fura-2-based calcium imaging experiments of stimulated with a mixture of OxPAPC and vitamin C (Vit. C; 10 mM, ◼) or OxPAPC (10 µM, ◼. HEK-293_TRPA1_: n = 5 per group, boxplot, median, t-test, p ≥ 0.05. (**c**) Analysis of the integrated density in HEK-293_TRPA1_ loaded with dihydroethidium 10, 20, and 30 min upon stimulation with carbonyl cyanide-*4*-(trifluoromethoxy) phenylhydrazone (FCCP; ◼, 10 µM) or OxPAPC (◼, 10 µM) compared to untreated control cells (◼, n = 3 with 18 cells per measurement, mean ± SEM, two-way ANOVA RM post hoc Holm-Sidak, p ≤  0.05.

**Supplementary Figure 7: AITC-evoked calcium responses in murine DRG neurons are not potentiated by subsequent OxPAPC stimulation and confirmation of data in rat DRG neurons.** Fura-2-based calcium imaging experiment: Increase in intracellular calcium in murine DRG (mDRG, **a**) and rat DRG (rDRG, **b-e**) neurons upon stimulation with AITC (▬, 10 µM), followed by OxPAPC (▬, 10 µM) stimulation (**a**) or vice versa (**b**). Stimulations of DRG neurons with capsaicin (▬, 1 µM) to activate TRPV1 and subsequent depolarization with 90 mM potassium ions (▬, K^+^) served as control stimuli. Isotype control antibody IgM (**c**, ▬, 1/300), E06 mAb (**d**, ▬, 1/300), or D-4F peptide (**e**, ▬, 100 µg/ml) were co-incubated with OxPAPC for 30 min at room temperature before stimulation of rDRGs. Agonists or mixtures were applied as indicated on the graph. Grey lines represent single cell responses; colored line shows mean of 50 single cell traces. Experiment represents one of six independent measurements. Ratio F(340/380): relative increase in fluorescence intensity (340/380 nm).

**Supplementary Figure 8: No hyperalgesia by E06 mAb injection alone and no anti-edematous effects in collagen-induced arthritis.** (**a**) Mechanical (paw pressure thresholds) and (**b**) thermal (paw withdrawal latencies) nociceptive thresholds were measured after injection of E06 mAb (1/100 = 0.01 µg; i.pl. ⭘) at indicated time points. (**c**) Edema (paw thickness) was measured in collagen-induced arthritis after treatment with E06 mAb or IgM isotype control Ab on day 13 after collagen-induced arthritis induction at the indicated time points. Mean values of n = 6 ± SEM, one-way ANOVA RM post hoc Holm-Sidak, p * ≤ 0.05.

**Supplementary Figure 9: No change in pain behavior in contralateral paws or in ipsilateral thermal hyperalgesia in rats treated with CFA or OxPAPC together with D-4F.** (**a, b**) Mechanical and thermal nociceptive thresholds (paw pressure threshold and paw withdrawal latency) were measured after injection of D-4F alone (2.5 mg/kg; i.pl. ⭘⚫). Time points of analysis are indicated. (**c, d**) Paw pressure threshold and paw withdrawal latency were determined in the contralateral paw at indicated time points after ipsilateral injection of D-4F (2.5 mg/kg i.pl. ⚫), OxPAPC (i.pl.) plus D-4F (◊, i.pl.) or OxPAPC intraplantarly plus D-4F systemically (◆,12.5 mg/kg i.p.) pre-administered for 5 d. (**e**) Paw withdrawal latency in contralateral paw was quantified after ipsilateral injection of CFA (⚫) or together with D-4F (12.5 mg/kg; i.p. ⚫) at indicated time points.

**Supplementary Figure 10: Acute antinociception after D-4F injection and reduction of inflammatory signs after D-4F in CFA-induced hindpaw inflammation or collagen-induced arthritis**. (**a**) Paw volume after ipsilateral injection of CFA (⚫), or CFA and D-4F (i.p.) (⚫) was determined. (**b-d**) Mechanical (paw pressure thresholds) nociceptive thresholds were measured on day 1-3 immediately and up to 3 h after treatment with D-4F (12.5 mg/kg i.p., ⚫) and compared to control rats without D-4F injections (⚫). (**e**) In collagen-induced arthritis, clinical scores were determined within one week after the first D-4F application (◼) compared to solvent treatment (🞏). (**a-d**) Mean values of n = 6 per group ± SEM, two-way ANOVA RM post hoc Holm-Sidak, p *, § ≤ 0.05. (**e**) n = 6 per group ± SEM, statistics.

**Reference**

1. Engel KM, Schroeter J. The Need for Commercially Available Defined Oxidized (Phospho)lipids*. Journal of Glycomics & Lipidomi*c**s** 06, (2016).

1. ^a^ The differentiation between triacylglycerols and hydroxylated triacylglycerols was made because CFA contains large amounts of castor oil, which contains considerable amounts of hydroxylated fatty acids. (*) Peaks at *m/z* 594.5 and 610.5 are derived from PAPC upon cleavage of the double bond next to the glycerol backbone. LPC, lysophosphatidylcholine; PC, phosphatidylcholine. [↑](#footnote-ref-2)
